# Quantum-classical mechanics as an alternative to quantum mechanics in molecular and chemical physics

**DOI:** 10.1016/j.heliyon.2019.e02579

**Published:** 2019-12-04

**Authors:** Vladimir V. Egorov

**Affiliations:** Russian Academy of Sciences, FSRC “Crystallography and Photonics”, Photochemistry Center, 7a Novatorov Street, Moscow, 119421, Russian Federation

**Keywords:** Theoretical physics, Quantum mechanics, Molecular quantum transitions, Singularity, Dozy chaos, Quantum-classical mechanics, Electron transfer, Condensed matter, Molecular physics, Optics, Applications

## Abstract

In quantum mechanics, the theory of quantum transitions is grounded on the convergence of a series of time-dependent perturbation theory. In nuclear and atomic physics, this series converges because the dynamics of quantum transitions (quantum jumps) are absent by definition. In molecular and chemical physics, on the contrary, the dynamics of “quantum” transitions, being determined by the joint motion of a light electron (or electrons) and very heavy nuclei, are present by definition, and the series of time-dependent perturbation theory becomes singular. An exception is the dynamic problem for stationary states in the Born-Oppenheimer adiabatic approximation, when the electronic subsystem turns out to be “off” from the general dynamic process and therefore is not dynamically full-fledged: it only forms an electric potential in which the nuclei oscillate. Removing the aforementioned singularity can be accomplished in two ways. The first method was consisted of introducing an additional postulate in the form of the Franck-Condon principle into molecular quantum mechanics, in which the adiabatic approximation is used. The second method was proposed by the author and consisted of damping the singular dynamics of the joint motion of an electron and nuclei in the intermediate (transient) state of molecular “quantum” transitions by introducing chaos. This chaos arises only during molecular quantum transitions and is called dozy chaos. Formally, the damping is carried out by replacing an infinitely small imaginary addition in the spectral representation of the complete Green's function of the system with its finite quantity. The damping chaos (dozy chaos) leads to the continuity of the energy spectrum in the molecular transient state, which is a sign of classical mechanics. Meanwhile, the initial and final states of the molecule obey quantum mechanics in the adiabatic approximation. Molecular quantum mechanics, which takes into account the chaotic dynamics of the transient state of molecular “quantum” transitions, can be called quantum-classical (dozy-chaos) mechanics. The efficacy of the damping for the aforementioned singularity was previously shown by dozy-chaos mechanics of elementary electron transfers in condensed matter, which is the simplest case of dozy-chaos mechanics, and its applications to a whole number of problems, especially to the optical spectra in polymethine dyes and their aggregates. This paper provides a regular exposition of this dozy-chaos (quantum-classical) mechanics of the elementary electron transfers. The main results of its applications presented in the introduction are also described.

## Teaser

Atomic physics; Condensed matter physics; Electromagnetism; Molecular physics; Optics; Quantum mechanics; Physics methods; Theoretical physics; Quantum mechanics; Molecular quantum transitions; Singularity; Dozy chaos; Quantum-classical mechanics; Electron transfer; Condensed matter; Applications

## 1 Introduction

### 1.1 A new theoretical approach to molecular quantum transitions and their applications

Quantum mechanics is one of the main, if not the most important, branches of modern theoretical physics. In the 20th century, atomic physics, nuclear physics, and solid state physics based on quantum mechanics created a fundamental basis for recent and modern technological progress. Therefore, even in a broad scientific environment, the possibilities of quantum mechanics are considered to be almost limitless. The applicability of quantum mechanics extends, for example, to the entire universe (see Refs. [1, 2, 3, 4]) and even the human brain and associated consciousness (see Refs. [5, 6, 7, 8, 9]). The word “quantum” is very fashionable in modern science (see Ref. [4]). In short, in the broad scientific community, there is a very popular view that quantum mechanics is the last and ultimate word about the essence of nature (see Ref. [9]). The current paper discusses the limits of the applicability of quantum mechanics based on an analysis of the internal contradictions that arise when it is applied unbiased to a wide range of problems in molecular and chemical physics.

Only a draft of the problem is outlined in the introduction. Later sections will include a detailed discussion on both the qualitative and quantitative levels. This section presents the main application results concerning the new approach proposed by the author in theory. This presentation differs from the generally accepted style, in which the theory is first presented and then its applications to the experiment are demonstrated. This is because most new results have already been published [10, 11, 12, 13, 14, 15, 16, 17, 18, 19] and reported at international conferences (see Refs. [20, 21, 22, 23, 24, 25, 26, 27, 28, 29, 30, 31, 32, 33, 34, 35, 36]). This article is based on publications and reports demonstrating the success of the new theory in applications to the experiment to discuss the new problem, that has arisen in physics, at a deeper physical level and provide a regular exposition of the theory in elementary electron transfers in condensed matter. There are two main reasons for simplifying this problem [11, 18]. The first involves approximating the electron's Green's function by it in the case of a free electron. The second is related to considering merely non-local vibrations of nuclei and disregarding their local vibrations.

A new theoretical approach to study of molecular quantum transitions and elementary electron transfers in condensed matter can be attributed accordingly to molecular and chemical physics. We call this new approach in theory dozy-chaos mechanics, or in another way, quantum-classical mechanics. The introduction of quantum-classical (dozy-chaos) mechanics into molecular and chemical physics has a forced character and is associated with the elimination of an essential singularity in the probabilities per unit time, or in other words, in the rate constants of quantum transitions in molecular systems. This singularity arises in quantum mechanics when it goes beyond the adiabatic approximation and follows from the incomparability of the masses of the electron (electrons) and nuclei and their joint motion in the process of molecular quantum transitions. The singularity is eliminated by changing the infinitesimal imaginary addition iγ in the spectral representation of the complete molecular Green's function with its finite quantity [10, 11, 12]. The matching of the new theory with experiments shows that the modulus of this imaginary addition γ is much more than the quantum of nuclear oscillations ℏω: γ>>ℏω [14, 28, 34]. The value of γ could be treated as the energy width of the electron-vibrational virtual levels of the transient state, which provides a multiple exchange of motion and energy between different vibrational modes of the nuclei and the electron in the transient state. However, because of the aforementioned excessively large value of γ, this exchange of motion and energy proves to be crash, and it causes chaos in the motion of both the nuclei and the electron that undergoes the transition. Since this chaos is absent in the initial and final states and present merely in the middle of molecular quantum transitions, it is called dozy chaos [14, 28, 33]. In chemical physics, the efficacy of the damping method for the aforementioned singularity was shown by the author with an example of the new (dozy-chaos) theory of elementary electron-charge transfers in condensed matter and its applications [10, 11, 12, 14, 15, 16, 17, 18, 19, 28, 29, 34, 35] to the optical spectra in polymethine dyes and their aggregates [37, 38, 39, 40, 41, 42, 43, 44] and its applications [20, 34, 45] to a number of other basic experimental data [46, 47].

The aforementioned quantum transitions in molecular and chemical physics (molecular quantum transitions), strictly speaking, are not quantum transitions, but quantum-classical transitions, since although their initial and final states are quantum, their transient states are of a classical nature. The quantum nature of the initial and final states is manifested in the fact that these states are often described by quantum mechanics in the adiabatic approximation and as a result of the reorganization of the nuclear subsystem, the structure of the final state of the molecular system differs markedly from the structure of its initial state. The latter fact is particularly evident for chemical reactions, which result in the formation of new molecules. The classical nature of the transient state of molecular systems is associated with the presence of chaos (dozy chaos) in the motion of electrons and nuclei involved in the quantum-classical transition, which leads to a continuous spectrum of their energies in this transient state [16, 18, 36, 48, 49]. The corresponding theory of quantum-classical transitions is called quantum-classical mechanics or dozy-chaos mechanics (see above). The aforementioned dozy-chaos theory of elementary electron-charge transfers in condensed matter is the simplest problem in quantum-classical mechanics^1^ and will be described in detail in subsequent sections.

Quantum-classical mechanics provide insights into an entire series of the fundamental experimental results in chemistry, which in the past often resisted insight in the scope of the standard quantum mechanics of electron-nuclear motion. In the framework of quantum-classical mechanics, it is possible to explain, for example, experimental data on the shape of the optical bands of polymethine dyes and their aggregates in solutions, in which the quantum-classical transitions in their main optical chromophores can be approximated by elementary electron-charge-transfer processes in condensed matter [10, 11, 12, 14, 15, 18, 28]. Exciton effects arising in many cases as a result of the aggregation of molecules somewhat complicate the overall picture of elementary quantum-classical transitions in dye chromophores, but do not fundamentally change it [10, 11, 12, 14, 15, 18, 28].

To model the electronic structure of the basic optical chromophore in polymethine dyes, their polymethine chain, Dähne [50] put forward the concept of an ideal polymethine state, according to which there is a clearly extended distribution of the density of the π-electron charge along the quasi-linear polymethine chain. This density varies periodically along the chain and reallocates alternately along it during optical excitation (see Ref. [51]). Moreover, for the first excited state, the moment of the electronic transition is focused on the chain [51]. Therefore, the elementary electron-charge transfer along the chain can approximate the electronic transition to the first excited state [10, 11, 12, 14, 15, 18, 28]. The polymethine dyes to be discussed herein can be considered as the ideal polymethine state of Dähne.

Since for an ideal polymethine state, the total transfer of the alternating charge along the full chain consists of the acts of elementary transfer of a small amount of charge over a small distance between adjacent carbon atoms, the tunneling effects in such an electron-charge transfer are minor and the Gamow tunnel factor is close to unity [10, 11, 12, 14, 15, 18, 28].

The linearity and sufficient length of the basic optical chromophores of polymethine dyes and their aggregates, associated with the linearity and a sufficiently large length of their polymethine chain, lead to the fact that we can neglect the interaction of the electronic transition with the motion of the nuclei of the dyes themselves and take into account only its interaction with environmental nuclei [10, 11, 12, 14, 15, 18, 28].

**Fig. 1:**
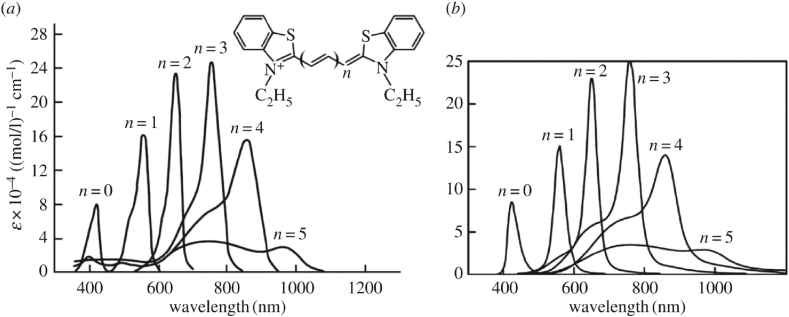
Experimental [41, 43] (*a*) and theoretical [12] (*b*) monomer's optical absorption spectra dependent on the length of the polymethine chain (thiapolymethinecyanine in methanol at room temperature; ε is the extinction coefficient) [15, 18, 19]. (Original citation) — Reproduced by permission of The Royal Society of Chemistry.

**Fig. 2:**
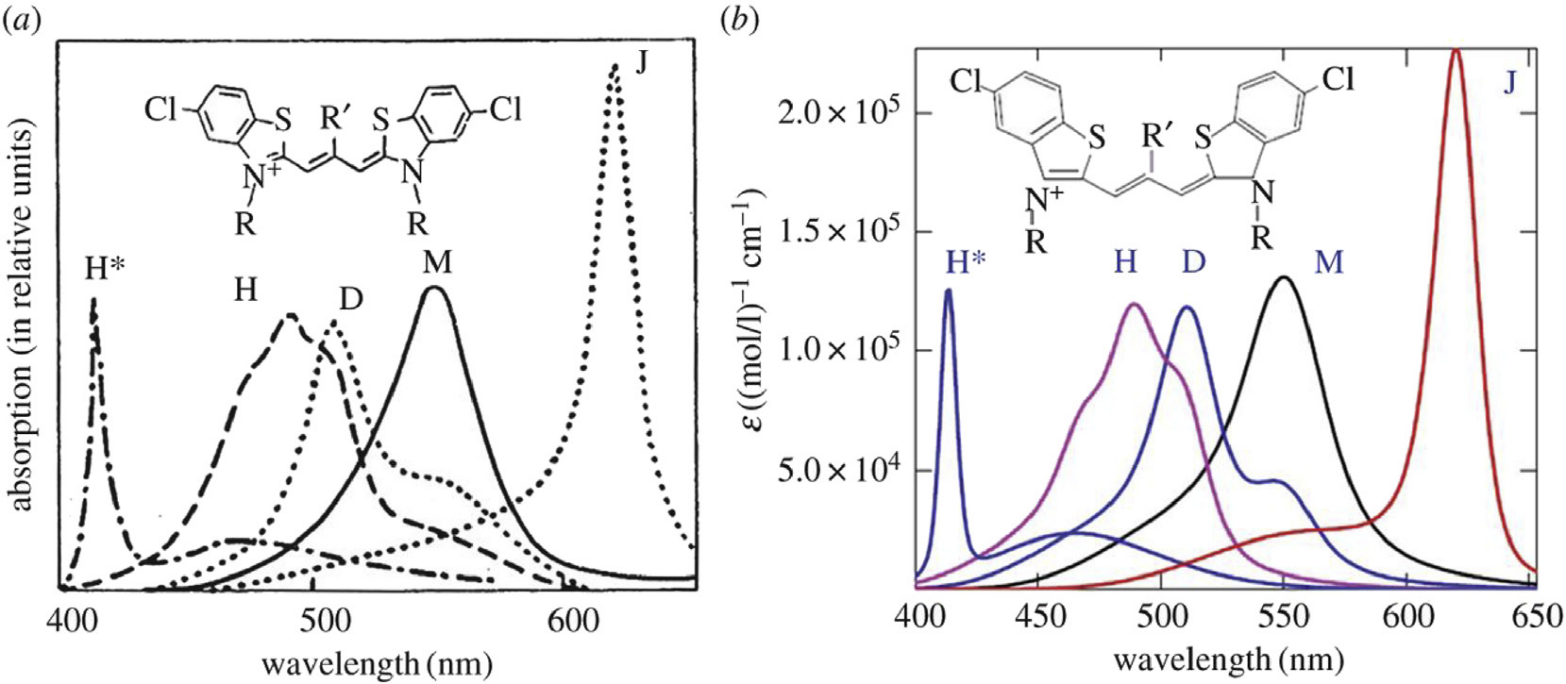
Theoretical optical absorption spectra [18, 19, 29, 35] (*b*) in thiapolymethinecyanines adjusted to the fundamental experimental data (*a*) on polymethine dye monomers (M), dimers (D), H-, H*-, and J-aggregates [43].

The most important results are in theoretical optical spectra adjusted by the author to the fundamental experimental data on polymethine dye monomers (M) [12, 14, 15, 18, 28, 41, 42, 43] (see Figs. 1 and 2), dimers (D) [15, 29, 42, 43], H and H* aggregates [16, 18, 29, 42, 43] (see Fig. 2), and J aggregates [10, 11, 12, 14, 17, 18, 28, 29, 37, 38, 39, 40, 42, 43] (see Fig. 2) and also theoretical spectra adjusted to the generally known data on the M–D [15, 29, 42, 43] and M–J aggregate [10, 11, 12, 14, 28, 42, 43] concentration equilibriums.

Among these important results, a special place is occupied by a theoretical explanation of the nature of the well-known narrow J band (see Fig. 2), which is determined by the dynamic pumping of an electronic transition in the chromophore of J aggregates (a tetramer with a brickwork-type structure) with weakly chaotic, or in other words, highly organized motion of nuclei in the environment (the case of weak dozy chaos) [10, 11, 12, 14, 18, 28]. The role of exciton effects in the shaping of the J band is insignificant. Conversely, the nature of the narrow H* band (see Fig. 2) is elucidated by the existence of dozy-chaos dynamics as well as a reasonably great exciton dynamics, their strong interference interaction [16, 18, 29]. A competitive interference of the chaotic dynamics of nuclear reorganization and exciton dynamics in the chromophore of the H * aggregate, which is the dimer (H * dimer), leads to a “pumping chaos” from the peak of the optical band of the H * dimer into its wing, making the peak even narrower and the wing still wider. This effect is associated with an abnormally strong exciton interaction in the H* dimer, which is a consequence of the specific structure of the H* monomers that make up it.

The H * monomers are cyclic bis-thiacarbocyanines that have an abnormally large area of a planar optical chromophore (see Ref. [42]). Therefore, there is a strong exciton interaction of the H * monomers in the H * dimer, which leads to the narrowing of the peak of its optical band as a result of “pumping chaos” from the peak into the wing of the band [16, 18, 29].

The striking results of quantum-classical mechanics are also the results of explaining the resonance behavior of the shape of the optical band of the polymethine dye monomer as a result of a change in the length of its polymethine chain (see Fig. 1) [12, 15, 18, 19, 41, 43] and a change in sign of the relative intensity of the two bands closest to the resonance band (see Fig. 1, n=3) as a result of a change in the polarity of the solvent [17, 18, 44]. Other dozy-chaos explanations can be also found in Ref. [17]. Dozy-chaos (quantum-classical) mechanics of “quantum” transitions in the discussed organic substance appears fairly sophisticated, but this substance in its facility in dynamical nature rates in quantum-classical mechanics like a hydrogen atom in structural nature in quantum mechanics [33b].

All of the results of the optical spectra match generally weak dozy chaos (γ<<E, where E is the so-called reorganization energy introduced in Section 2.4.1). Strong dozy chaos (γ≥E) leads to the elucidation of the important patterns in the reactions of proton transfer [45, 47] and comparatively fresh temperature-dependent effects on electron transfers in Langmuir-Blodgett films [20, 46]. In the case of strong dozy chaos, the dynamics of quantum-classical transitions become weakly dependent on dozy chaos, and the electronic component of the complete electron-nuclear amplitude of transitions can be fitted by the Gamow tunnel exponential dependent on the transient phonon environment. This elementary method permit us to evade the consideration of the imaginary additive iγ in the spectral representation of the complete Green's function and to word the physical nature of the transient state not in the concept of dozy chaos, but in the concept of a large number of tunnel and over-barrier energy states providing the “quantum” transition of an elementary charged particle. This method was worked out [52] long before the development of quantum-classical mechanics [11, 12, 14, 15, 18, 28] (see the subsequent sections), and now we can say that the concept of a large number of tunnel and over-barrier states is the simplest version of the concept of dozy chaos.

Grounded on this method [52], in 1990 a theoretical description of the basic experimental patterns in the Brönsted relationships [47] for the reactions of proton transfer (acid-base catalysis) was given [45]. The Brönsted relationships was found by Brönsted and Pedersen in 1924 (see Ref. [47]). The theory in Ref. [52] is immediately appropriate to the explanation of electron transfers. To explain the reactions of transfers of heavy charged particles (proton transfers), the result of thermic fluctuations of the potential barrier transparence must be considered because of fluctuations of the barrier width. In contrast to the elementary proton transfer, the electron transfer process is insensitive to small fluctuations of the barrier width due to the large size of the electronic wave function in the initial and final states. The analytical formulas for the proton-transfer rate constants are obtained. In acid catalysis, the Brönsted relationship is $\mathrm{lg}K^{\left(\text{acid}\right)}=\alpha \;\mathrm{lg}K_{\text{eq}}+a$, where $K^{\left(\text{acid}\right)}$ is the rate constant, $K_{\text{eq}}$ is the equilibrium constant, and α and a are constants. In base catalysis, the Brönsted relationship is $\mathrm{lg}K^{\left(\text{base}\right)}=\beta \;\mathrm{lg}K_{\text{eq}}+b$. The theoretically obtained Brönsted coefficients α and β for direct and inverse reactions meet the generally known empirical equality α+β=1. The experimentally ascertained large extent of linearity of the Brönsted relationships is elucidated by the concept of a large number of tunnel and over-barrier states, and by the phonon frequencies dispersion and the barrier width fluctuations. The kinetic isotope effect (proton, deuteron or triton transfer) computed using our theory is in agreement with experimental data (see Ref. [45]). In some cases (see Ref. [45]) the regularities of acid-base reactions are coupled not with the proton transfers, but with the electron transfers. The criteria for selecting one or another mechanism of reactions are formulated in Ref. [45]. By considering the generally known similarity of the relationships of Brönsted and Tafel's law in electrochemistry, the theory of acid-base catalysis can be adapted to electrochemical reactions [45].

Ref. [20] provides an explanation for the temperature dependence of the activation energy $E_{\text{a}}=E_{0}\left(1-T/T_{0}\right)$ (where $T_{0}\approx 350\text{ K}$) for electron transfers at high temperatures in Langmuir-Blodgett films, which is discovered by Naito and Miura [46], as well as an interpretation of the entire set of these authors' experimental data in a wide diapason of temperatures. The activation energy $E_{\text{a}}$ reduces and proves to be negative as temperature T grows because the domain of tunnel states expands on the energy scale and nears the apex of the electron potential barrier, and the quantity of over-barrier states grows.

Section 2.1 considers fundamental questions of the theory of molecular quantum transitions.

## 2 Main text

### 2.1 Nature of molecular quantum transitions and the concept of dozy chaos

At the end of the 19th century, most physicists believed that the physical picture of the world was complete or nearing completion. This physical picture of the world was based on classical mechanics. However, the theoretical description of the equilibrium radiation of an absolutely black body, the problem of the so-called ultraviolet catastrophe, and the constancy of the velocity of light and its independence from the motion of the reference frame remained to be elucidated. The theory of relativity and quantum mechanics emerged as a result.

Quantum mechanics soon led to the development of atomic and nuclear physics. Quantum mechanics was also the basis for molecular physics, which rapidly developed in two directions. The first was associated with the theory of the structure of molecules and solids, which was based on the Born-Oppenheimer adiabatic approximation [53]. The second was associated with molecular optical spectroscopy based on the Franck-Condon principle [54, 55, 56, 57]. The adiabatic approximation, in which the motion of light electrons quickly adapts to the slow motion of heavy nuclei and which often perfectly describes the stationary (ground) state of molecular systems, is in full compliance with the principles of quantum mechanics. The Franck-Condon principle assumes that during optical excitation of a molecule, a light electron makes a fast quantum jump (transition) to an excited state, and then the whole configuration of heavy nuclei in the molecular system slowly adapts to the new distribution of the electron charge in the excited state. This assumption leads in many cases to a good agreement between theoretical and experimental results of molecular optical spectra.

Unlike in atomic spectroscopy, where there is no physical meaning, with a few exceptions,^2^ in molecular spectroscopy, the dynamics of a quantum transition are of considerable significance and are already determined by the Franck-Condon principle.^3^ Although the Franck-Condon dynamics of the quantum transition cause a number of *a priori* physical objections,^4^ due to the good agreement of the theory with experiments, for almost 100 years it has been an “unshakable” physical postulate within molecular quantum mechanics.

Thus, the generally accepted theory of molecular quantum transitions has a long history based on the well-known classical works of Born and Oppenheimer [53] and Franck and Condon [54, 55, 56, 57], which appeared immediately after the development of the foundations of quantum mechanics. Born and Oppenheimer were the first to solve the quantum-mechanical problem for a system of coupled electrons and nuclei in the simplest case for molecules [53], offering an effective and successful physical and mathematical technique, later called adiabatic approximation. Adiabatic approximation originated from adiabatic invariants, which were previously formulated by Ehrenfest [60]. The effectiveness of adiabatic approximation results from the Born-Oppenheimer theory of a significant difference in the masses of electrons and nuclei, which enables the separation of the slow motion of very heavy nuclei from the rapid motion of light electrons by neglecting the small nonadiabaticity operator in the Schrödinger equation to solve quantum-mechanical problems. The Born-Oppenheimer theory [53] is the basis of modern solid-state physics and quantum chemistry.

A completely different picture forms in connection with the works of Franck and Condon [54, 55, 56, 57], the basis of the so-called Franck-Condon principle. As previously mentioned, due to the significant difference in the masses of electrons and nuclei, the dynamics of molecular quantum transitions consists of two stages. In the first stage, under the influence of an external perturbation, a light electron nearly instantaneously passes into an excited electronic quantum state. In the second stage, a so-called reorganization of the nuclear subsystem of the molecule occurs, in which the spatial configuration of the heavy nuclei slowly adapts to the new electron-charge distribution. However, such a picture raises a serious objection. Namely, due to the significant inertia of the nuclei, they will not follow the movement of the electron, but simply return the electron to its original state just as rapidly [14, 28, 33b]. In other words, proceeding from these general considerations, the probability (per unit time) of a molecular quantum transition must be zero. However, if the heavy nuclei suddenly begin to move after a light electron already in the excited state, then due to the substantial inertia of the nuclei, they cannot stop in the excited electronic state [14, 28, 33b]. In other words, the probability (per unit time) of a molecular quantum transition must be infinite. Thus, these simple and general qualitative considerations demonstrate that there is a singularity in the rates of the molecular quantum transitions. At one time, these thoughts were either not discussed or considered, apparently because of the success of the Franck-Condon principle in its practical applications, which resulted in the development of modern molecular optical spectroscopy. Nevertheless, the question of the validity of the Franck-Condon principle remained unchanged in theory. In this regard, the Franck-Condon principle should not be treated as a physical principle, but as an efficient hypothesis that does not have a thorough theoretical justification.

In addition to the previously mentioned qualitative objections, there is no formal quantitative substantiation for the Franck-Condon principle in theory. Proceeding from the first principles of quantum mechanics, it is necessary to quantitatively solve the problem of the dynamics of the transient state in molecular quantum transitions, at least in some simple cases. Moreover, such a solution, particularly substantiating the Franck-Condon principle, should clearly be demonstrated in many diverse applications. However, such a solution has not been obtained to date.

Returning to the qualitative physical consideration: “Under what condition is a light electron able to move nuclei with substantial inertia and thereby allow the reorganization of the nuclear subsystem in molecular quantum transitions?” [14, 28, 33b]. The electron should abandon attempts to move the nuclei “alone” but somehow provoke the same nuclei to move. How does this occur if the nuclei in the molecule have at least vibrational motion and at all temperatures up to absolute zero (zero-point vibrations)? To shift the equilibrium positions of oscillating nuclei in space, their vibrational motions must be transformed, at least in part, into the translational motion of their equilibrium positions during the molecular quantum transition. This is easy to implement if the motions of the nuclei are chaotic during the molecular quantum transition. If the vibrations of the nuclei are chaotic, an electron could easily control their motion during “quantum” transition. As shown by the formal theory, which will be discussed later, electrons that bind nuclei (atoms) to molecules cause chaotic motion in nuclei in a transient state. In other words, a light electron, through the creation of chaos, organizes the motion of very heavy nuclei during molecular “quantum” transition, resulting in this transition (that is, it occurs at a non-zero and non-infinite rate as previously mentioned). This is the essence of the self-organization of molecular quantum transitions. This occurs through the creation of chaos by an electron in a transient molecular state. This is called dozy chaos as discussed in Section 1. Dozy chaos is the joint effect of the collective chaotic motion of electrons and nuclei, and their chaotic electromagnetic interactions in the transient state of molecules experiencing quantum transitions. Dozy chaos is a universal physical phenomenon, because electrons and nuclei are universal [14, 15, 16, 18, 28, 32, 33, 34, 35, 36, 48, 49].

One can cite some *a priori* objection to the previously mentioned qualitative reasoning, from which it follows that there is a singularity (that is, zero or infinity) in the rates of molecular transitions. This objection is that the nuclei, even because of their substantial inertia, cannot return an excited light electron to its original ground state since the electron is already in a well-defined quantum state from which it cannot escape because of the presence of an energy gap in the electronic energy according to quantum mechanics. Therefore, in the excited state, the electron is forced to slowly adjust the nuclear subsystem to its new charge distribution. This constitutes the essence of the Franck-Condon principle. However, our *a priori* objection and the Franсk-Condon principle itself are entirely based on the *a priori* assumption that quantum mechanics works in this case. As previously mentioned, this assumption has no formal evidence. Moreover, qualitative considerations about the continuous energy spectrum in the transient state, which appears due to the electron provocation of chaos in the vibrational motion of the nuclei to control their motion in this state (as previously mentioned), indicate that in reality there is no gap in the electronic energy, and consequently, quantum mechanics ceases to work in a transient dynamic molecular state. In other words, the excited electron in principle cannot be “hooked” into the quantum state, which follows from the standard solution to Schrödinger's equation, and therefore it quickly “rolls” over the continuous spectrum of energy to the ground state, as discussed in our initial qualitative reasoning.

Considering quantum mechanics as applied to molecular quantum transitions, according to the Franck-Condon principle, in optical excitation the electron transition occurs at the turning points of the oscillating nuclei, that is, at the moment they stop. This “reasonable assumption” in quantum mechanics^5^ makes it possible to “switch off” the nuclear subsystem at this time from a dynamic analysis, just as in the framework of quantum mechanics the electronic subsystem is “switched off” from a full-fledged dynamic analysis in a justified procedure to isolate the nonadiabaticity operator in Schrödinger's equation and neglecting it in the adiabatic approximation (see Section 2.3).

The Franck-Condon principle essentially follows from two physical facts. The first assumes (in general, unreasonable, as previously noted) that quantum mechanics works in the field of molecular quantum transitions. The second assumes that, over the course of their classical oscillations in an oscillatory potential well, the nuclei spend a majority of the time near the turning points (see footnote 5). Therefore, the quantum transition (see the first fact) has an overwhelming probability of occurring at these points, and consequently, it will be vertical or close to vertical. Thus, the same oscillator behaves in the same elementary molecular way both as a classical system (near the bottom of an oscillatory potential well for nuclei, where the probability of a quantum transition is negligible) and as a quantum system (near the turning points of the nuclei, where the probability of a quantum transition is maximal). Therefore, the oscillator is essentially a quantum-classical system (see also Section 2.5), and the Franck-Condon principle is essentially one of the most important prerequisites for the creation of quantum-classical mechanics. During the formulation of the Franck-Condon principle [54, 55, 56, 57], the prospect of creating quantum-classical mechanics was not noticed, apparently because of the strongest “psychological confrontation” between quantum and classical mechanics.

The time of the classical behavior of the oscillator near its bottom (or, equivalently, far from the turning points of the nuclei) is much shorter than the period of the oscillations of the nuclei. This correlates with the fact in quantum-classical mechanics that the time of molecular “quantum” transition or, equivalently, the time of reorganization of the nuclear subsystem ℏ/E (E is the energy of the reorganization of the nuclear subsystem as mentioned in Section 1), when, due to chaos in the transient state, the electron-nuclear system leads like the classical system (see above), is also much shorter than the nuclear oscillation period $\frac{\hbar }{E}<<ℑ$, where E=(1÷0.1) eV and $ℑ≅10^{-13}\text{s}$ ($\left(10^{-15}÷10^{-14}\right)\text{ s}\;<<10^{-13}\text{ s}$).

The dynamics of the transient state in molecular quantum transitions can be approached from the other side, namely, from the characters of the optical spectra observed in the experiment. The discrete line spectrum in atoms is a direct indication of the existence of quantum jumps in atoms. The nature of molecular spectra differs from that of atomic spectra: they are striped or often even continuous, as for example in the case of polymethine dyes and their aggregates (see Section 1). The continuity of the spectra, observed for polymethine dyes and their aggregates, indicates that the transition from one quantum state to another is not a quantum jump but a transition through a continuous spectrum of electron-vibrational states.^6^ (The continuity of the spectra is also associated with the dispersion of the phonon frequencies; see Section 2.9).

In strong dozy chaos (γ≥E), quantum-classical mechanics are compatible with the Born–Oppenheimer [53] and Franck–Condon [54, 55, 56, 57] quantum mechanics with high precision [11, 12, 14, 15, 16, 18, 28, 34] (see Sections 2.4.9, 2.5, and 2.9). It turns out that in this particular case, the rates of “quantum” transitions, as in standard molecular quantum mechanics, depend only on the initial and final states, since due to strong dozy chaos they do not depend on the dynamics of the transient state. In other words, dozy chaos often “leaves no trace” in molecular physics, which is why it took so long to discover it. In these often practiced cases, standard molecular quantum mechanics gives results that are consistent with the experiment, despite the fact that they are based on erroneous concepts [11, 12, 14, 15, 16, 18, 28, 34]. In other words, the Franck-Condon principle is an effective simulator of strong dozy chaos, both in the problem of elementary electron transfers in condensed matter, and, presumably, in all other cases of molecular “quantum” transitions (see details in Section 2.5).

Extremely strong dozy chaos (γ>>E) leads to standard molecular quantum mechanics, but with abnormally low rates of “quantum” transitions. Weak dozy chaos (γ<<E) causes a strong dynamic self-organization of molecular “quantum” transitions and hence their high rates [11, 12, 14, 15, 16, 18, 28, 34] (see Section 1).

### 2.2 Divergence of a series of time-dependent perturbation theory in quantum mechanics for molecular quantum transitions

Turning to formal techniques of quantum mechanics, we can precisely indicate the point at which quantum mechanics applied to molecular quantum transitions ceases to work. As is well known, the theory of quantum transitions is based on the time-dependent perturbation theory (TDPT) in quantum mechanics (see *e.g.* Ref. [61]). The time-dependent Schrödinger equation is solved by the standard scheme of the perturbation theory [61]:

(1) $$i\hbar \frac{\partial \Psi }{\partial t}=\text{H}\left(t\right)\Psi ,\;\text{H}\left(t\right)={\text{H}}_{0}+V\left(t\right),\;V\left(t\right)=\left\{ \begin{array}{l} W\left(t\right),\;if\;0\leq t\leq \tau ,\\ \text{0, }if\;t<0,\;t>\tau \end{array}\right..$$

The series for the transition amplitude $A_{fl}$ is as follows

(2) $$A_{fl}\left(t\right)=\sum_{n=0}^{\infty }\frac{\langle f|I_{n}\left(t\right)|l\rangle }{n!},$$

where

(3) $$I_{n}\left(t\right)={\left(\frac{1}{i\hbar }\right)}^{n}P\int_{0}^{t}dt_{1}\int_{0}^{t}dt_{2}...\int_{0}^{t}dt_{n}\tilde{W}\left(t_{1}\right)\tilde{W}\left(t_{2}\right)...\tilde{W}\left(t_{n}\right),$$

P is the Dyson's chronological operator and

(4) $$\tilde{W}\left(t\right)=e^{\frac{i}{\hbar }{\text{H}}_{0}t}W\left(t\right)e^{-\frac{i}{\hbar }{\text{H}}_{0}t}$$

is the perturbation operator in the interaction representation. The series of TDPT converges in atomic and nuclear physics, as in the matrix elements of transitions $\langle f|I_{n}\left(t\right)|l\rangle$ in the amplitude (2), due to quantum jumps, the dynamics of quantum transitions is not contained by definition, and for many problems it suffices to confine oneself to the first order of TDPT. The probability of a quantum transition from state |l〉 to state |f〉 during the time of the perturbation τ is determined by the formula (“Fermi's golden rule”, see Ref. [61])

(5) $${ℑ}_{fl}\left(\tau \right)={\left|A_{fl}^{\left(1\right)}\left(\tau \right)\right|}^{2}=\frac{1}{\hbar^{2}}{\left|\int_{0}^{\tau }\langle f|\tilde{W}\left(t\right)|l\rangle e^{i\omega_{fl}t}dt\right|}^{2},$$

where $\hbar \omega_{fl}=E_{f}-E_{l}$, $E_{f}$ and $E_{l}$ are the energies of stationary states.

The series of TDPT converges also in the case of molecular quantum transitions (and electronic transitions in condensed matter), which occur in accordance with the Franck-Condon principle [54, 55, 56, 57], that is, when the states |l〉 and |f〉 in the amplitude (2) are taken in the Born-Oppenheimer adiabatic approximation [53]. In other words, the physical picture of quantum transitions in molecular physics is usually represented as quantum jumps, which are similar to quantum jumps in atomic and nuclear physics. (A small parameter that ensures the convergence of the series is a small perturbation time τ, see Eq. (1).) The resulting theoretical (Franck-Condon [54, 55, 56, 57]) transition probabilities (per unit time) that follow from Eq. (5) often explain the experimental data well, for example, in molecular spectroscopy (see *e.g.* Refs. [62, 63, 64]). However, in chemistry, for example, there are many experimental data (see *e.g.* Section 1), which can not be explained within the adiabatic approximation. This forces us to go beyond the adiabatic approximation. But such an exit beyond the adiabatic approximation leads to divergence of the series of TDPT in quantum mechanics, which is associated with the incomparability of the masses of electrons and nuclei, jointly participating in the dynamics of the transition, which, unlike to atomic and nuclear transitions, is contained here by definition. To verify this fact, the divergence of the series of TDPT is enough to demonstrate by any one example. As such an example, it is convenient to consider the simplest problem in the theory of quantum transitions, which goes beyond the adiabatic approximation. Such a simple problem, as it turned out, is the problem of the theoretical description of the elementary electron transfers in condensed matter [11, 18]. As previously mentioned (Section 1 and footnote 1), the simplicity of this problem is related to the sufficiency of taking into account only the non-local phonons in the matter and the possibility of approximating the electron Green's function by the propagator (see Sections 2.4.7 and 2.4.4 below) [11, 18].

Before proceeding the formulation of this simplest theory, that is, a new theory of elementary electron transfers in condensed matter, the author believes that for its better understanding it is important to remind the reader of the essence of the Born-Oppenheimer adiabatic theory.

### 2.3 The Born-Oppenheimer adiabatic approximation^7^

The standard base for a general study of electron-vibrational interactions is the adiabatic theory [53, 65, 66, 67], which uses the only universal small parameter of the molecule — the Born-Oppenheimer parameter $\kappa ={\left(\mu /\text{M}\right)}^{1/4}$, where μ is the electron mass and M is the typical mass of the nucleus.

The Hamiltonian $\hat{H}$ of a molecule is written as the sum of the kinetic energy of electrons ${\hat{T}}_{e}$ and nuclei ${\hat{T}}_{N}$ and the total potential energy $U\;\left(q,p\right)$ of the molecule:

(6) $$\hat{H}={\hat{T}}_{e}+{\hat{T}}_{N}+U\left(q,p\right),$$

where q and p are the set of electronic and nuclear coordinates. In the adiabatic approximation the wave function $\Psi \left(q,p\right)$ of the molecule is searched in the form

(7) Ψ(q,p) = ψ(q,p) ϕ(p).

Substituting Eq. (7) into the stationary Schrӧdinger equation

(8) $$\hat{H}\Psi \left(q,p\right)=E\Psi \left(q,p\right),$$

where the Hamiltonian $\hat{H}$ is given by Eq. (6), we obtain

(9) $$\phi {\hat{T}}_{e}\psi \;+\;{\hat{T}}_{N}\psi \phi \;+\;U\psi \phi \;=\;E\psi \phi .$$

We formally introduce the operator $\hat{L}$ defined from the equation:

(10) $$\hat{L}\psi \phi \;=\;{\hat{T}}_{N}\psi \phi \;-\;\psi {\hat{T}}_{N}\phi .$$

Substituting ${\hat{T}}_{N}\psi \phi$ from Eq. (10) into Eq. (9) and dividing both sides of the equation by ψϕ, we get

(11) $$\frac{1}{\psi }{\hat{T}}_{e}\psi \;+\;U\;+\;\frac{1}{\psi \phi }\hat{L}\psi \phi \;=\;E\;-\;\frac{1}{\phi }{\hat{T}}_{N}\phi \text{.}$$

Let us mark the right part of this equation, which depends solely on the nuclear coordinates p, through V(p); then from Eq. (11) and this notation we obtain

(12) $$\left({\hat{T}}_{e}\;+\;U+\;\frac{1}{\phi }\hat{L}\phi \right)\;\psi \;=\;V\psi \text{,}$$

(13) $$\left({\hat{T}}_{N}\;+\;V\right)\;\phi \;=\;E\phi \text{.}$$

Eqs. (12) and (13) are identical to the original Schrödinger Eq. (8). It is only rewritten for the new formally introduced functions ψ(q,p) and ϕ(p) in Eq. (7).

The adiabatic approximation agrees with the disregard for the term $\left(1/\phi \right)\hat{L}\phi$ in Eq. (12), which is called the nonadiabaticity operator. Hence the system of adiabatic equations has the form

(14) $$\left[{\hat{T}}_{e}\;+\;U\left(q,p\right)\right]\;\psi_{f}\;=\;V_{f}\left(p\right)\;\psi_{f}\text{,}$$

(15) $$\left[{\hat{T}}_{N}\;+\;V_{f}\left(p\right)\right]\;\phi_{fn}\;=\;E_{fn}\;\phi_{fn}\text{.}$$

The solution of the electron Schrödinger Eq. (14) gives a system of electron wave functions and energy levels that depend on the nuclear coordinates p as parameters. The electron energy $V_{f}\left(p\right)$ acts as a potential function for the motion of the nuclei (see Eq. (15)). This function is often called the electron term. The concept of electron term or potential energy surface (PES) plays a key role not only in the theory of molecules and quantum chemistry as a whole, but also in the modern theory of elementary chemical reactions and elementary processes of electron-charge transfers. If the use of PESs in the former case is perfectly justified, then in the latter case, when it comes to quantum transitions in the PES intersection area (in the transient region), the Born-Oppenheimer (BO) adiabatic approximation does not work at all. This can be seen if we write out the corrections to the BO approximation in perturbation theory with respect to the nonadiabaticity operator:

(16) $$V_{f}^{\left(1\right)}=\langle \psi_{f}^{\left(0\right)}|{\hat{T}}_{N}|\psi_{f}^{\left(0\right)}\rangle \text{,}$$

(17) $$V_{f}^{\left(2\right)}=\sum_{f^{\prime }\neq f}\frac{{\left|\langle \psi_{f^{\prime }}^{\left(0\right)}|{\hat{T}}_{N}|\psi_{f}^{\left(0\right)}\rangle -\sum_{p}\frac{\hbar^{2}}{\text{M}}\frac{1}{\phi }\frac{\partial \phi }{\partial p}\langle \psi_{f^{\prime }}^{\left(0\right)}|\frac{\partial \psi_{f}^{\left(0\right)}}{\partial p}\rangle \right|}^{2}}{V_{f}^{\left(0\right)}-V_{f^{\prime }}^{\left(0\right)}}$$

and

(18) $$\psi_{f}^{\left(1\right)}=\sum_{f^{\prime }\neq f}\frac{\langle \psi_{f^{\prime }}^{\left(0\right)}|{\hat{T}}_{N}|\psi_{f}^{\left(0\right)}\rangle -\sum_{p}\frac{\hbar^{2}}{\text{M}}\frac{1}{\phi }\frac{\partial \phi }{\partial p}\langle \psi_{f^{\prime }}^{\left(0\right)}|\frac{\partial \psi_{f}^{\left(0\right)}}{\partial p}\rangle }{V_{f}^{\left(0\right)}-V_{f^{\prime }}^{\left(0\right)}}\cdot \psi_{f^{\prime }}^{\left(0\right)}\text{.}$$

The BO approximation is violated for the electron energy in the second order of perturbation theory and for the electron wave function in the first order of perturbation theory. It can be seen that the nonadiabatic corrections $V_{f}^{\left(2\right)}$ from Eq. (17) and $\psi_{f}^{\left(1\right)}$ from Eq. (18) are not minor in the case when the differences of the electronic terms $V_{f}^{\left(0\right)}-V_{f^{\prime }}^{\left(0\right)}$, which depend on the nuclear coordinates p and which enter into the denominators, turn out to be small.

Thus, the PESs, being, as a rule, dynamic (adiabatic) invariants for the initial and final states of the electron-nuclear system,^8^ are not dynamic invariants for the transient state. Therefore, the problem of finding dynamical invariants for the transient state arises. We will solve this problem in the simplest case of “chemical transformations”: in the case of extended electron-vibrational transitions or, in other words, in the case of elementary electron transfers in condensed matter.

### 2.4 Quantum-classical mechanics of elementary electron transfers in condensed matter

#### 2.4.1 Adiabatic approximation for elementary electron transfers

In comparison with the Hamiltonian in the standard theory of many-phonon transitions (see Ref. [72]), in the theory of elementary electron transfers the Hamiltonian is complicated merely by a complementary electron potential well $U_{2}\left(q-L\right)$ set apart from the original well $U_{1}\left(q\right)$ by the distance L≡|L| [11]:

(19) $$\text{H}=-\frac{\hbar^{2}}{2\mu }\Delta_{q}+U_{1}\left(q\right)+U_{2}\left(q-L\right)+\sum_{\iota }U_{\iota }\left(q\right)\;p_{\iota }+\frac{1}{2}\sum_{\iota }\hbar \omega_{\iota }\left(p_{\iota }^{2}-\frac{\partial^{2}}{\partial p_{\iota }^{2}}\right)\text{,}$$

where μ is the effective mass of an electron, q is the electron's radius vector, $p_{\iota }$ are the normal phonon coordinates (real), $\omega_{\iota }$ are the eigenfrequencies of normal oscillations, and ι is the phonon index; $\sum_{\iota }U_{\iota }\left(q\right)\;p_{\iota }$ is the electron–phonon coupling expression.

In the adiabatic approximation, the solution of the Schrödinger equation

(20) $$\text{H }\Psi =E_{\text{H}}\;\Psi$$

for the system “electron + environment” is sought in the form

(21) Ψ(q,p) = ψ(q,p) Φ(p)

(compare with Eq. (7) in Section 2.3), where the electron ψ-function satisfies the Schrödinger equation

(22) $$\left[-\frac{\hbar^{2}}{2\mu }\Delta_{q}+U_{1}\left(q\right)+U_{2}\left(q-L\right)+\sum_{\iota }U_{\iota }\left(q\right)p_{\iota }\right]\psi \left(q,p\right)=E\left(p\right)\psi \left(q,p\right)\text{.}$$

If we neglect the nonadiabaticity operator (compare with Eq. (10) in Section 2.3)

(23) $$\hat{L}\Psi \equiv {\hat{T}}_{p}\psi \Phi -\psi {\hat{T}}_{p}\Phi =-\sum_{\iota }\hbar \omega_{\iota }\left(\frac{\partial \psi }{\partial p_{\iota }}\frac{\partial \Phi }{\partial p_{\iota }}+\frac{\Phi }{2}\frac{\partial^{2}\psi }{\partial p_{\iota }^{2}}\right)$$

$\left({\hat{T}}_{p}=-\frac{1}{2}\sum_{\iota }\hbar \omega_{\iota }\frac{\partial^{2}}{\partial p_{\iota }^{2}}\right)$ in Eq. (20), then

(24) $$\left[E\left(p\right)+\frac{1}{2}\sum_{\iota }\hbar \omega_{\iota }\left(p_{\iota }^{2}-\frac{\partial^{2}}{\partial p_{\iota }^{2}}\right)\right]\Phi \left(p\right)=E_{\text{H}}^{\text{BO}}\Phi \left(p\right)\text{,}$$

where $E_{\text{H}}^{\text{BO}}$ is the approximate eigenvalue of the total Hamiltonian (19). The index BO in Eq. (24) and beyond indicates that the corresponding quantity is accepted in the Born-Oppenheimer adiabatic approximation.

The electronic Eq. (22) is solved by perturbation theory with the perturbation operator

(25) $$\tilde{U}\equiv \sum_{\iota }U_{\iota }\left(q\right)\left(p_{\iota }-{\tilde{p}}_{\iota }\right)\text{,}$$

similar to the perturbation operator of Pekar [73, 74]. It will be shown below that ${\tilde{p}}_{\iota }$ are the values of the normal phonon coordinates for which the adiabatic potential in Eq. (24) has a minimum.

In the zero order of the perturbation theory with respect to the operator $\tilde{U}$, for the electron Eq. (22) we have

(26) $$\left[-\frac{\hbar^{2}}{2\mu }\Delta_{q}+U_{1}\left(q\right)+U_{2}\left(q-L\right)+\sum_{\iota }U_{\iota }\left(q\right)\;{\tilde{p}}_{\iota }\right]\psi_{s}\left(q\right)=E_{s}^{0}\psi_{s}\left(q\right)\text{,}$$

where the index s numbers the states of the discrete and continuous spectra. It is assumed that the interaction

$$\sum_{\iota }U_{\iota }\left(q\right)\;{\tilde{p}}_{\iota }$$

at the donor 1 and at the acceptor 2 is the same, that is, by definition

(27) $${\tilde{p}}_{\iota 2}=-{\tilde{p}}_{\iota 1}\equiv -{\tilde{p}}_{\iota }\;\left({\tilde{p}}_{\iota }<0\right)$$

and

(28) $$U_{\iota 2}\left(q-L\right)=-U_{\iota 1}\left(q\right)\equiv -U_{\iota }\left(q\right)\text{.}$$

In this wise

$$\sum_{\iota }U_{\iota 2}\left(q-L\right){\tilde{p}}_{\iota 2}=\sum_{\iota }\left[-U_{\iota 1}\left(q\right)\right]\left[-{\tilde{p}}_{\iota 1}\right]=\sum_{\iota }U_{\iota 1}\left(q\right){\tilde{p}}_{\iota 1}\equiv \sum_{\iota }U_{\iota }\left(q\right){\tilde{p}}_{\iota }\text{.}$$

The first order correction to the electron energy $E_{s}^{0}\left(\tilde{q}\right)$ is

(29) ∫dqψs∗(q)U˜(q,p)ψ(q)s=∫dqψs∗(q)[∑ιUι(q)(pι−p˜ι)]ψs(q)=∑ιUιs(pι−p˜ι),

where

(30) $$U_{\iota s}\equiv \int dqU_{\iota }\left(q\right){\left|\psi_{s}\left(q\right)\right|}^{2}\text{.}$$

Thus, in the first order of perturbation theory with respect to the operator $\tilde{U}$, for the electron energy we have

(31) $$E_{s}\left(p\right)=E_{s}^{0}+{\tilde{U}}_{s}\left(p\right),\;{\tilde{U}}_{s}\left(p\right)\equiv \sum_{\iota }U_{\iota s}\left(p_{\iota }-{\tilde{p}}_{\iota }\right)\text{.}$$

So, in the presence of an electron (on a donor or on an acceptor), the operator of nuclear energy in the adiabatic approximation has the form:

(32) $$E_{s}\left(p\right)+\frac{1}{2}\sum_{\iota }\hbar \omega_{\iota }\left(p_{\iota }^{2}-\frac{\partial^{2}}{\partial p_{\iota }^{2}}\right)=E_{s}^{0}+\sum_{\iota }U_{\iota s}\left(p_{\iota }-{\tilde{p}}_{\iota }\right)+\frac{1}{2}\sum_{\iota }\hbar \omega_{\iota }\left(p_{\iota }^{2}-\frac{\partial^{2}}{\partial p_{\iota }^{2}}\right)\text{.}$$

The role of the potential energy of nuclei is played by the function

(33) $$F_{s}\left(p\right)=E_{s}^{0}+\sum_{\iota }U_{\iota s}\left(p_{\iota }-{\tilde{p}}_{\iota }\right)+\frac{1}{2}\sum_{\iota }\hbar \omega_{\iota }p_{\iota }^{2}\text{.}$$

We find its minimum:

$$\frac{dF_{s}}{dp_{\iota }}=U_{\iota s}+\hbar \omega_{\iota }p_{\iota },\;\frac{dF_{s}}{dp_{\iota }}{|}_{p_{\iota }={\bar{p}}_{\iota }}=0,\;\frac{d^{2}F_{s}}{dp_{\iota }^{2}}=\hbar \omega_{\iota }>0\text{;}$$

(34) $${\bar{p}}_{\iota }=-\frac{U_{\iota s}}{\hbar \omega_{\iota }}\text{,}$$

where $U_{\iota s}$ are given by Eq. (30). Suppose that $U_{\iota }\left(q\right)\approx \text{constant}$. We choose this constant in the following form: $\text{constant}\equiv -\hbar \omega_{\iota }{\tilde{p}}_{\iota }$. Then, taking

$${\int dq\left|\psi_{s}\left(q\right)\right|}^{2}=1\text{,}$$

we obtain

(35) $${\bar{p}}_{\iota }={\tilde{p}}_{\iota }\text{,}$$

that is, in the aforementioned assumption, ${\tilde{p}}_{\iota }$ are the values of the normal phonon coordinates at which the adiabatic potential of Eq. (24) for the nuclear wave function Φ(p) has a minimum. Thus, the function $U_{\iota }\left(q\right)$ is assumed to be equal to its average over the electron state in the zero order of perturbation theory with respect to the operator $\tilde{U}$.

The minimum value of the potential energy of the nuclei in Eq. (33) is

(36) $$F_{s}\left(p\right){|}_{p=\tilde{p}}=E_{s}^{0}+\frac{1}{2}\sum_{\iota }\hbar \omega_{\iota }{\tilde{p}}_{\iota }^{2}\equiv E_{s}^{0}+E\equiv J_{s}\text{,}$$

where

(37) $$E\equiv \frac{1}{2}\sum_{\iota }\hbar \omega_{\iota }{\tilde{p}}_{\iota }^{2}$$

is the energy of reorganization of the nuclear vibrations due to the presence of an electron on the donor or the acceptor. The potential energy of the nuclei is written in terms of the quantities $J_{s}$ in the form

(38) $$F_{s}\left(p\right)=J_{s}+\frac{1}{2}\sum_{\iota }\hbar \omega_{\iota }{\left(p_{\iota }-{\tilde{p}}_{\iota }\right)}^{2}\text{.}$$

In the two-level approximation $J_{s}\equiv -J_{1,2}<0$: in the initial state the electron is on the donor 1, and in the final state — on the acceptor 2. The corresponding potential energy surfaces of the nuclei $F_{1,2}\left(p\right)$ are the two paraboloids of dimension M, where M is the number of vibrational degrees of freedom of the crystal, the vertices of which, according to Eq. (27), are displaced relative to each other by the amount $2\left|\tilde{p}\right|$. The operator of the total energy of nuclei (Eq. (32)) has the form

(39) $$-J_{1,2}\;+\;\frac{1}{2}\;\sum_{\iota }\;\hbar \omega_{\iota }\;\left[{\left(p_{\iota }-{\tilde{p}}_{\iota }\right)}^{2}-\frac{\partial {\;}^{2}}{\partial \;p_{\iota }^{2}}\right]\text{.}$$

Its eigenvalues are equal to

(40) $$E_{\text{H}}^{\text{BO}}=-J_{1,2}+\sum_{\iota }\hbar \omega_{\iota }\left(m_{\iota 1,2}+\frac{1}{2}\right)\text{,}$$

and its eigenfunctions are equal to

$$\Phi_{1,2...m_{\iota 1,2}...}\left(p-\tilde{p}\right)=\prod_{\iota }\varphi_{m_{\iota 1,2}}\left(p_{\iota }-{\tilde{p}}_{\iota }\right),$$

(41) φmι1,2(pι−p˜ι)=Amι1,2e−12(pι∓p˜ι)2Hmι1,2(pι∓p˜ι).

Here $H_{m}\left(p\right)={\left(-1\right)}^{m}e^{p^{2}}d^{m}e^{-p^{2}}/dp^{m}$ are the Hermite polynomials of degree m (m=0,1,2,...), $A_{m}$ is the normalizing factor.

Thus, for the wave function of the “electron + environment” system in the Born-Oppenheimer adiabatic approximation (Eq. (21)) and according to Eq. (26), we have

(42) $$\Psi_{1,2}^{\text{BO}}\left(q,p\right)=\psi_{1,2}\left(q\right)\Phi_{1,2...m_{\iota 1,2}...}\left(p-\tilde{p}\right)\text{.}$$

An adiabatic approximation of the type Eq. (42), in which the electron wave function ψ does not depend on the nuclear coordinates p, is often called the rough adiabatic approximation.

#### 2.4.2 Technique of Green's functions

In accordance with our aim to discover “successful” dynamic invariants for the transient state [12, 13], which would be alternative to the Born-Oppenheimer adiabatic invariants — potential energy surfaces, we search for the solution of the Schrödinger Eq. (20) for the “electron + environment” system by Green's function technique. At the initial stage of constructing this solution, the identical transformations of Eq. (20) in the Green's function method can be considered as alternatives to identical transformations when the non-adiabaticity operator (23) is singled out in the Born-Oppenheimer method (see Section 2.4.1): if the purpose of the latter is to separate the slow motion nuclei from the rapid motion of an electron, the purpose of the former is to maximally preserve the interconnection of electronic and nuclear movements.

So, to the Hamiltonian of the “electron + environment” system of some general form H=H(q,p), we add and subtract some, while arbitrary, operator $\tilde{U}=\tilde{U}\left(q,p\right)$:

(43) $$\text{H}\equiv \text{H}-\tilde{U}+\tilde{U}\text{.}$$

Then the Schrödinger Eq. (20) can be rewritten in the form

(44) $$\left(\text{H}-\tilde{U}-E_{\text{H}}\right)\;\Psi =-\tilde{U}\;\Psi \text{.}$$

We consider the right-hand side in Eq. (44) as an inhomogeneity. The corresponding homogeneous equation has the form

(45) $$\left(\text{H}-\tilde{U}-E_{\text{H}}\right)\;\tilde{\Psi }=0\text{.}$$

Let us introduce the Green's function $G_{\text{H}}$ and G:

(46) $$\left(\text{H}-E_{\text{H}}\right)G_{\text{H}}\left(q,q^{\prime };p,p^{\prime };E_{\text{H}}\right)=-\Delta \left(q-q^{\prime }\right)\Delta \left(p-p^{\prime }\right)\text{,}$$

(47) $$\left(\text{H}-\tilde{U}-E_{\text{H}}\right)G\left(q,q^{\prime };p,p^{\prime };E_{\text{H}}\right)=-\Delta \left(q-q^{\prime }\right)\Delta \left(p-p^{\prime }\right)\text{.}$$

They are connected by the Dyson integral equation

(48) $$G_{\text{H}}\left(q,q^{\prime };p,p^{\prime };E_{\text{H}}\right)=G\left(q,q^{\prime };p,p^{\prime };E_{\text{H}}\right)+\iint dq_{1}dp_{1}G\left(q,q_{1};p,p_{1};E_{\text{H}}\right)\tilde{U}\left(q_{1},p_{1}\right)G_{\text{H}}\left(q_{1},q^{\prime };p_{1},p^{\prime };E_{\text{H}}\right)$$

(this can be easily verified by acting the operator $\left(\text{H}-\tilde{U}-E_{\text{H}}\right)$ on the left and right sides of the equation). In a symbolic way, the Dyson equation (Eq. (48)) has the form

(49) $$G_{\text{H}}=G+G\tilde{U}G_{\text{H}}\text{.}$$

The solution of Eq. (49) is found by successive approximations

(50) $$G_{\text{H}}\;=\;G\;+\;G\;\tilde{U}\;G\;+\;G\;\tilde{U}\;G\;\tilde{U}\;G\;+\;...\text{.}$$

The general solution of Eq. (44) for the wave function Ψ(r,q) of the “electron + environment” system has the form

(51) $$\Psi \left(q,p\right)=\tilde{\Psi }\left(q,p\right)+\iint dq^{\prime }dp^{\prime }G\left(q,q^{\prime };p,p^{\prime };E_{\text{H}}\right)\tilde{U}\left(q^{\prime },p^{\prime }\right)\Psi \left(q^{\prime },p^{\prime }\right)\text{.}$$

This integral equation is the Lippmann-Schwinger equation. It is identical to the original Schrödinger Eq. (20) or (44) (this can be easily verified by acting the operator $\left(\text{H}-\tilde{U}-E_{\text{H}}\right)$on the left and right sides of the equation and taking into account Eq. (45)). We rewrite the Lippmann-Schwinger equation (Eq. (51)) in the symbolic form

(52) $$\Psi =\tilde{\Psi }+G\tilde{U}\Psi \text{.}$$

We find its solution by successive approximations

(53) $$\begin{array}{c} \Psi \;=\;\tilde{\Psi }\;+\;G\;\tilde{U}\;\tilde{\Psi }\;+\;G\;\tilde{U}\;G\;\tilde{U}\;\tilde{\Psi }\;+\;G\;\tilde{U}\;G\;\tilde{U}\;G\;\tilde{U}\;\tilde{\Psi }\;+\;...\\ =\tilde{\Psi }\;+\;\left(G\;+\;G\;\tilde{U}\;G\;+\;G\;\tilde{U}\;G\;\tilde{U}\;G\;+\;...\right)\;\tilde{U}\;\tilde{\Psi }\text{.} \end{array}$$

Taking into account the solution of the Dyson equation for $G_{\text{H}}$ (see Eq. (50)), we obtain

(54) $$\Psi =\tilde{\Psi }+G_{\text{H}}\;\tilde{U}\;\tilde{\Psi }\text{.}$$

This wave function Ψ is a formal solution of the original Schrödinger Eq. (20) or (44). Our next problem is, by a proper choice of the operator $\tilde{U}$ in the solution (54), to separate the state of elementary transfer of the electron, which we are interested in, from nonphysical solutions corresponding to other types of electron and nuclear motions. To do this, it is necessary to specify the Hamiltonian H=H(q,p) of the “electron + environment” system, which figures while in some general form in our Green's function method.

In the simplest case, we have Hamiltonian (19), which was already used by us earlier in considering the Born-Oppenheimer adiabatic approximation. We choose the operator $\tilde{U}$, that figures in our method of Green's functions, in the form of the operator (25), which we used earlier (Section 2.4.1) in the Born-Oppenheimer method:

(25a) $$\tilde{U}=\sum_{\iota }U_{\iota }\left(q\right)\left(p_{\iota }-{\tilde{p}}_{\iota }\right)\text{.}$$

Here, in contrast to Eq. (25), ${\tilde{p}}_{\iota }$ are some, so far arbitrary, constants. Then

(55) $$\text{H}-\tilde{U}=-\frac{\hbar^{2}}{2\mu }\Delta_{q}+U_{1}\left(q\right)+U_{2}\left(q-L\right)+\sum_{\iota }U_{\iota }\left(q\right)\;{\tilde{p}}_{\iota }+\frac{1}{2}\sum_{\iota }\hbar \omega_{\iota }\left(p_{\iota }^{2}-\frac{\partial^{2}}{\partial p_{\iota }^{2}}\right)$$

and the solution of the homogeneous Eq. (45),

(56) $$\tilde{\Psi }\left(q,p\right)=\psi \left(q\right)\Phi_{0}\left(p\right)\text{,}$$

corresponds to the lack of interaction of the electron with the vibrations of the nuclei. In other words, the nuclei vibrate as if there were no electron in the condensed medium, and the motion of the electron occurs as if the nuclei were immovably fixed in their equilibrium positions. Thus, the solution $\tilde{\Psi }\left(q,p\right)$ does not correspond to either the electron-transfer state or many-phonon transitions in general. Therefore, it must be excluded from the general solution (54) as describing an unphysical state. Consequently, the state of electron transfer is determined by the solution [79].

(57) $$\Psi =G_{\text{H}}\;\tilde{U}\;\tilde{\Psi }\text{.}$$

#### 2.4.3 Electron-phonon wave functions of the initial and final states. On the convergence of a series of time-dependent perturbation theory in quantum-classical mechanics

In the solution (57), however, the fact of the “start” of the electron from the donor 1 is not yet reflected. This fact is taken by substituting the wave function in the adiabatic approximation (42) into account in the right-hand side of Eq. (57), which corresponds to the presence of interaction of the electron with the vibrations of the nuclei, instead of the wave function (56), which corresponds to the lack of this interaction. Formally, this reduces only to a shift in the normal phonon coordinates by an amount $\tilde{p}$ in the phonon part of the wave function. As a result, the constants ${\tilde{p}}_{\iota }$ introduced above (Eq. (25a)) acquire the physical meaning of the shifts of the normal phonon coordinates, which correspond to the shifts in the equilibrium positions of the nuclei, caused by the presence of an electron in the medium. In other words, the quantities ${\tilde{p}}_{\iota }$ in the method of Green's functions have the same physical meaning as in the Born-Oppenheimer method. Thus, the state of electron transfer at its “start” from the donor 1 is described by the wave function [79].

(58) $$\Psi_{1}=G_{\text{H}}\;\tilde{U}\;\Psi_{1}^{\text{BO}}\text{.}$$

We recall that in the solution $G_{\text{H}}$ (see Eq. (50)) of the Dyson Eq. (49) the Green's function G is determined by the Hamiltonian (55) with the separating variables q and p. So, in the Green's function $G_{\text{H}}$ it remains to find only the value of the total energy $E_{\text{H}}$ of the system. This energy is taken in the adiabatic approximation corresponding to the wave function $\Psi_{1}^{\text{BO}}$ (see Eqs. (24) and (40, 41, 42)). As a result, we obtain the wave function (58), where the Green's function $G_{\text{H}}=G_{\text{H}}\left(E_{\text{H}}=E_{\text{H}}^{\text{BO}}\right)$ is expressed in terms of $G=G\left(E_{\text{H}}=E_{\text{H}}^{\text{BO}},\;i\gamma \to 0\right)$ as a series (50).

The infinitesimal imaginary additive iγ specified in G is ordinarily written in the spectral representation of the Green's function in order to escape zero in the energy denominator. In our case, the spectral representation of the Green's function has the form:

(59) G≡G(q,q′; p,p′; H−U˜) = ∑s Ψs(q,p) Ψs∗(q′,p′)EHBO−(Es(p)−U˜(p)s)−iγ,

where $\Psi_{s}\left(q,p\right)$ are the eigenfunctions of the Hamiltonian (55) and $E_{\text{H}}^{\text{BO}}=E_{s}\left(p\right)$ (see Section 2.4.1); the energy denominator vanishes when ${\tilde{U}}_{s}\left(p=\tilde{p}\right)=0$ (${\tilde{U}}_{s}\left(p\right)\equiv \sum_{\iota }U_{\iota s}\left(p_{\iota }-{\tilde{p}}_{\iota }\right)$, see Eq. (31)) and γ=0. We, however, ascribe a finite value to the value of γ, as a result of which γ acquires the physical meaning of the degree of chaos in the reorganization of the nuclei of the medium, provoked by the motion of the electron from the donor towards the acceptor.^9^ Thus, for the elementary electron transfers, along with the energy of reorganization $E\equiv \frac{1}{2}\sum_{\iota }\hbar \omega_{\iota }{\tilde{p}}_{\iota }^{2}$ (see Eq. (37)) of the nuclei of a medium, a dissipation energy γ is introduced into the theory, which characterizes the degree of chaos in the motion of nuclei in a transient state. The implementation of the finite quantity γ makes it possible to escape the singularity in the rates of elementary electron transfers, which is formally associated with the vanishing of the energy denominator of the complete Green's function of the system at γ=0, and which is physically associated with the incomparability of the masses of the electron and nuclei of the environment,^10^ and it indicates physically insertion, to the transient state, of a process for the transformation, at first, of a portion of the vibrational motion of the nuclei into their translational motion, and after that, in the course of completing the “quantum” transition, of the emerged translational motion of the nuclei back into their vibrational motion. Since the chaos in the electron-nuclear movement is absent in the initial and final states and occurs merely in the transient state, it is called ***dozy chaos***, and the appropriate dissipation energy γ can be called the ***dozy-chaos energy*** [14, 28].

Along with the objective of damping the singular electron-nuclear movement, our procedure of introducing a finite magnitude of γ has extra important objective: it allows us to ensure the parameter of smallness of the problem [79].

(60) $$\tilde{U}\;G∼\frac{\tilde{U}}{\gamma }∼\frac{\hbar \omega_{\iota }}{\gamma }<<1\text{.}$$

Hence

(61) $$G>>G\;\tilde{U}\;G>>G\;\tilde{U}\;G\;\tilde{U}\;G>>...\text{,}$$

and in obedience to the series (50), the Green's function $G_{\text{H}}$ is as follows

(62) $$G_{\text{H}}\approx G\text{.}$$

Thus, in accordance with Eq. (58), we finally have a solution describing the state of electron transfers:

(63) $$\Psi_{1}\approx G\;\tilde{U}\;\Psi_{1}^{\text{BO}}\text{,}$$

where $G=G\left(E_{\text{H}}=E_{\text{H}}^{\text{BO}};\;i\gamma ,\;\gamma >>\hbar \omega_{\iota }\right)$.

Starting from relationship (60), it is not difficult to understand that the wave function of the system for an electron localized on the acceptor 2, in order to avoid nullity in the transition amplitude

(64) $$A_{12}=\langle \Psi_{2}\left(q-L,p\right)|\text{W}|\Psi_{1}\left(q,p\right)\rangle \text{,}$$

should be taken no longer in the form of Eq. (63), but in the adiabatic approximation (Eq. (42)): $\Psi_{2}=\Psi_{2}^{\text{BO}}$.^11^

The series for the transition rate constant, which corresponds to the Green's function series (Eq. (50)), according to relationship (60) has a small parameter

(65) $${\left({\bar{m}}_{1}\hbar \omega_{\iota }/\gamma \right)}^{2}<<1\text{,}$$

where ${\bar{m}}_{1}$ is the distribution function of Planck.^12^ Therefore, in our problem for $k_{\text{B}}T>\hbar \omega_{\iota }/2$, as a small parameter, we have

(66) $${\left(k_{\text{B}}T/\gamma \right)}^{2}<<1\text{.}$$

So, according to Eq. (60), the parameter for the convergence of the Dyson series for the Green's function $G_{\text{H}}$ (Eq. (50)) is

(67) $$\frac{\hbar \omega }{\gamma }<<1$$

(for simplicity, we assume the vibrational frequency ω=ιconstant≡ω). Therefore, the same parameter (Eq. (67)) ensures the convergence of the TDPT series (see Section 2.2) for the amplitude of the quantum transitions $A_{fl}\left(t\right)$ (see Eq. (2)), in which the initial (l) and final (f) states are determined by the regular wave functions previously obtained: the initial state — by the dynamic wave function according to Eq. (63), and the final state — by the wave function taken in the adiabatic approximation (Eq. (42)).

#### 2.4.4 The simplest electron-phonon Green's function

In Eq. (63), the Green's function $G\left(q,q^{\prime };L;p,p^{\prime };E_{\text{H}}^{\text{BO}}\right)$ is given by the expression following from its spectral representation (59) and the form of the Hamiltonian (55) with the separating variables q and p:

(68) $$G\left(q,q^{\prime };L;p,p^{\prime };E_{\text{H}}^{\text{BO}}\right)=\sum_{...m_{\iota }...}G_{e}\left(q,q^{\prime };L;\tilde{p};E_{\text{H}}^{\text{BO}}-\varepsilon_{...m_{\iota }...}\right)\Phi_{0...m_{\iota }...}\left(p\right)\Phi_{0...m_{\iota }...}\left(p^{\prime }\right)\text{,}$$

where

(69) $$G_{e}\left(q,q^{\prime };L;\tilde{p};E_{\text{H}}^{\text{BO}}-\varepsilon_{...m_{\iota }...}\right)=\sum_{s}\frac{\psi_{s}\left(q;L;\tilde{p}\right)\psi_{s}*\left(q^{\prime };L;\tilde{p}\right)}{\left(E_{\text{H}}^{\text{BO}}-\varepsilon_{...m_{\iota }...}\right)-E_{s}\left(L;\tilde{p}\right)-i\gamma }$$

is the Green's function of the extended electron motion associated with the virtual motion of the nuclei. It follows from the form of the Hamiltonian (55) with separating variables q and p that the electronic wave functions $\psi_{s}\left(q;L;\tilde{p}\right)$ in Eq. (69) are the eigenfunctions of Eq. (26),^13^ and the phonon wave functions $\Phi_{0...m_{\iota }...}\left(p\right)$ in Eq. (68) are given by Eq. (41) for $\tilde{p}=0$ and replacing the indices of the initial and final states $1,2...m_{\iota 1,2}...$ by the index of intermediate states $0...m_{\iota }...$. The phonon energies in Eq. (69) are

(70) $$\varepsilon_{...m_{\iota }...}\equiv \sum_{\iota }\hbar \omega_{\iota }\left(m_{\iota }+\frac{1}{2}\right)$$

(compare with Eq. (40) in Section 2.4.1).

The general expression for the electron Green's function (Eq. (69)) has the form (see Ref. [80])

(71) $$G_{e}=G^{\left(\text{free}\right)}-G^{\left(\text{free}\right)}\hat{T}G^{\left(\text{free}\right)}\text{,}$$

where

(72) $$G^{\left(\text{free}\right)}\equiv G^{\left(\text{free}\right)}\left(q,q^{\prime };k\right)=\frac{\mu }{2\pi \hbar^{2}}\frac{\exp\left(\pm ik\left|q-q^{\prime }\right|\right)}{\left|q-q^{\prime }\right|}$$

is the propagator or the Green's function of the free motion of an electron with energy $\hbar^{2}k^{2}/2\mu$, and $\hat{T}$ denotes the T-operator (scattering operator). In our case, in the denominator of $G_{e}$ (see Eq. (69)), according to Eqs. (40) and (70), we have the electron energy

(73) $$E_{\text{H}}^{\text{BO}}-\varepsilon_{...m_{\iota }...}-i\gamma =-\left(J_{1}+\hbar \omega_{1}+i\gamma \right)\text{,}$$

where

(74) $$\omega_{1}\equiv \sum_{\iota }\omega_{\iota }\left(m_{\iota }-m_{\iota 1}\right)\text{.}$$

From here

(75) $$k=\pm i{\left[2\mu \left(J_{1}+\hbar \omega_{1}+i\gamma \right)\right]}^{1/2}/\hbar \text{.}$$

In the problem under consideration, the properties of the T-operator are determined by the form of the electron potential wells $U_{1}\left(q\right)$ and $U_{2}\left(q-L\right)$. We choose these potentials in the simplest form, namely, in the form of the zero-radius Fermi potentials [81, 82]. It is clear that in this case, for not too small distances |L|≡L, which we have for the transfer of the electronic charge, the term $G^{\left(\text{free}\right)}\hat{T}G^{\left(\text{free}\right)}$ in Eq. (71) does a significant part just in such small surroundings of points q=0 and q=L (see Ref. [83]), which give a small contribution to the transfer rate constant [11, 79]. Therefore, for $G_{e}$ (see Eq. (69)) we have

(76) $$G_{e}≅G^{\left(\text{free}\right)}\text{.}$$

Taking into account Eqs. (72) and (75), we obtain

(77) $$G_{e}\left(q,q^{\prime };\alpha \right)=\frac{\mu }{2\pi \hbar^{2}}\frac{\exp\left(-\alpha \left|q-q^{\prime }\right|\right)}{\left|q-q^{\prime }\right|}\text{,}$$

where

(78) $$\alpha \equiv \alpha \left(...\;m_{\iota }\;...\right)\equiv \alpha \left(\omega_{1}\right)={\left[2\mu \left(J_{1}+\hbar \omega_{1}+i\gamma \right)\right]}^{1/2}/\hbar$$

($\omega_{1}$ is given by the formula (74)). Thus, substituting $G_{e}$ from Eq. (77) into Eq. (68), for the simplest electron-phonon Green's function we have

(79) $$G\left(q,q^{\prime };p,p^{\prime }\right)=\sum_{...m_{\iota }...}G_{e}\left[q,q^{\prime };\alpha \left(...\;m_{\iota }\;...\right)\right]\Phi_{0...m_{\iota }...}\left(p\right)\Phi_{0...m_{\iota }...}\left(p^{\prime }\right)\text{,}$$

where the electron Green's function $G_{e}\left[q,q^{\prime };\alpha \left(...\;m_{\iota }\;...\right)\right]$ is given by Eq. (77), and the phonon wave functions $\Phi_{0...m_{\iota }...}\left(p\right)$ are given, as previously indicated, by Eq. (41) for $\tilde{p}=0$ and replacing the indices of the initial and final states $1,2...m_{\iota 1,2}...$ by the index of intermediate states $0...m_{\iota }...$.

#### 2.4.5 The simplest Green's function of elementary electron transfers

As a result of neglecting the T-operator term in Eq. (71), the simplest electron-phonon Green's function (Eq. (79)) does not depend on the distance L between the donor and the acceptor of the electron charge. The dependence on the distance L arises after integration over coordinates q of the electron in the transfer amplitude (Eq. (64)). Therefore, the definition of the simplest Green's function for the elementary electron-charge transfers must include this integration.

Substituting the expression (58) for the wave function $\Psi_{1}\left(q,p\right)$ written in detail in terms of the Green's function according to Eqs. (68) and (69) and expression (42) for the wave function $\Psi_{2}\left(q-L,p\right)$ in the adiabatic approximation into the amplitude given by Eq. (64), we obtain

(80) $$A_{12}=\sum_{l}\sum_{...m_{\iota }...}A_{l}\left[J_{1}+\sum_{\iota }\hbar \omega_{\iota }\left(m_{\iota }-m_{\iota 1}\right);L;\tilde{p}\right]\frac{o\left(m_{l},m_{l1}\right)}{r\left(m_{l},m_{l1}\right)}\prod_{\iota }r\left(m_{\iota 2},m_{\iota }\right)r\left(m_{\iota },m_{\iota 1}\right)$$

where (taking into account Eq. (35))

(81) $$A_{l}\left[J_{1}+\sum_{\iota }\hbar \omega_{\iota }\left(m_{\iota }-m_{\iota 1}\right);L;\tilde{p}\right]=-\hbar \omega_{l}{\tilde{p}}_{l1}\iint dqdq^{\prime }G_{e}\left[q,q^{\prime };\alpha \left(...\;m_{\iota }\;...\right)\right]\psi_{2}*\left(q\right)\left|q-L\right|\psi_{1}\left(q^{\prime }\right)\text{,}$$

(82) $$\prod_{\iota }r\left(m_{\iota },m_{\iota 1,2}\right)\equiv \prod_{\iota }\int \varphi_{m_{\iota }}\left(p_{\iota }\right)\varphi_{m_{\iota 1,2}}\left(p_{\iota }-{\tilde{p}}_{\iota }\right)dp_{\iota }=\int \Phi_{0...m_{\iota }...}\left(p\right)\Phi_{1,2...m_{\iota 1,2}...}\left(p-\tilde{p}\right)dp,$$

(83) $$o\left(m_{l},m_{l1}\right)\equiv -\int \varphi_{m_{l1}}\left(p_{l}^{\prime }-{\tilde{p}}_{l}\right)\left(p_{l}^{\prime }-{\tilde{p}}_{l}\right)\varphi_{m_{l}}\left(p_{l}^{\prime }\right)dp_{l}^{\prime },$$

where, for their part, $\varphi_{...}\left(...\right)$ are the wave functions of the linear harmonic oscillator (Eq. (41) for $\tilde{p}=0$ and/or $\tilde{p}\neq 0$). The factor |q−L| in the integrand of Eq. (81) comes from the perturbation operator W in the transition amplitude (Eq. (64)), which is accepted in the long-wave approximation (see Section 2.4.3).

Using the zero-radius approximation [81, 82] for the electron potential wells $U_{1}\left(q\right)$ and $U_{2}\left(q-L\right)$ (see Section 2.4.4), for the wave functions $\psi_{1}\left(q^{\prime }\right)$ and $\psi_{2}*\left(q\right)$ in Eq. (81) we have

(84) $$\psi_{1}\left(q^{\prime }\right)={\left(\frac{\alpha_{1}}{2\pi }\right)}^{1/2}\frac{\exp\left(-\alpha_{1}\left|q^{\prime }\right|\right)}{\left|q^{\prime }\right|},$$

(85) $$\psi_{2}*\left(q\right)={\left(\frac{\alpha_{2}}{2\pi }\right)}^{1/2}\frac{\exp\left(-\alpha_{2}\left|q-L\right|\right)}{\left|q-L\right|},$$

where

(86) $$\alpha_{1,2}=\frac{{\left[2\mu \left(J_{1,2}-E\right)\right]}^{1/2}}{\hbar }\text{,}$$

and E is the energy of reorganization of the vibrations of the nuclei (see Eq. (37)), $J_{1}>J_{2}>E$ by definition. Substituting $G_{e}\left[q,q^{\prime };\alpha \left(...\;m_{\iota }\;...\right)\right]$ from Eq. (77) and $\psi_{1}\left(q^{\prime }\right)$ and $\psi_{2}*\left(q\right)$ from Eqs. (84) and (85) into formula (81) and computing the integral $\int dq^{\prime }...$, we get

(87) $$A_{l}=-\frac{\mu {\left(\alpha_{1}\alpha_{2}\right)}^{1/2}}{{\left(2\pi \hbar \right)}^{2}}\hbar \omega_{l}{\tilde{p}}_{l1}\int I\left(\left|q\right|\right)\exp\left(-\alpha_{2}\left|q-L\right|\right)dq,$$

where

(88) $$I\left(\left|q\right|\right)=\frac{4\pi \left[\exp\left(-\alpha \left|q\right|\right)-\exp\left(-\alpha_{1}\left|q\right|\right)\right]}{\left(\alpha_{1}^{2}-\alpha^{2}\right)\left|q\right|}.$$

As a result, after computing the integral ∫...dq in Eq. (87), we have [11, 79].

(89) $$A_{l}=c_{12}\omega_{l}{\tilde{p}}_{l1}G^{\text{E}}\text{,}$$

where

(90) $$c_{12}=-\frac{8\mu \alpha_{2}{\left(\alpha_{1}\alpha_{2}\right)}^{1/2}}{\hbar }\text{,}$$

the Green's function of the elementary electron transfers is

(91) $$G^{\text{E}}=G^{\text{E}}\left(\alpha ,L\right)=\frac{1}{L}\left[\frac{\exp\left(-\alpha L\right)+f\left(\alpha ,L\right)}{\left(\alpha_{1}^{2}-\alpha^{2}\right){\left(\alpha_{2}^{2}-\alpha^{2}\right)}^{2}}\right]\text{,}$$

(92) $$f\left(\alpha ,L\right)\equiv \left\{\left[1+\frac{\left(\alpha_{2}^{2}-\alpha_{1}^{2}\right)L}{2\alpha_{2}}\right]\exp\left(-\alpha_{2}L\right)-\exp\left(-\alpha_{1}L\right)\right\}\frac{{\left(\alpha_{2}^{2}-\alpha^{2}\right)}^{2}}{{\left(\alpha_{2}^{2}-\alpha_{1}^{2}\right)}^{2}}-\left[\frac{L}{2\alpha_{2}}\exp\left(-\alpha_{2}L\right)\right]\left(\alpha_{2}^{2}-\alpha^{2}\right)-\exp\left(-\alpha_{2}L\right)\text{,}$$

and the function $\alpha =\alpha \left(\omega_{1}\right)$ is given by formula (78). Thus, the Green's function of the electron-charge transfers $G^{\text{E}}=G^{\text{E}}\left(\omega_{1},L\right)$ to the distance L is given by Eqs. (91) and (92), in which the function $\alpha =\alpha \left(\omega_{1}\right)$ is given by Eq. (78). The values of $\hbar \omega_{1}$, on which α depends, are the energy levels of the electron in the transient state, which arise as a result of virtual movements of the electron and nuclei.

#### 2.4.6 The general expression for the rate constant of elementary electron photo-transfers. The technique of generating functions

Taking into account the quasi-discreteness of the phonon energy spectrum in a condensed matter having a bounded volume, we introduce an energy unit $\hbar \bar{\omega }$ so small that all the phonon energies $\hbar \omega_{\iota }$ are expressed by integers [72, 76, 77]. The heat energy of elementary electron-charge transfers is then given by an integer $\Delta \equiv \hbar \omega_{12}>0$.^14^ Then, proceeding according to the rules of quantum mechanics, within the “Golden Fermi rule”, the rate constant of the elementary electron-charge photo-transfers (optical extinction) ε for the $\omega_{12}$-phonon act can be formulated in the following way [72, 73, 74]:

(93) $$\varepsilon =\frac{4\pi^{2}q^{2}M_{\text{A}}\Omega }{3\hbar cn_{\text{ref}}}K$$

where q is the quantity of electron charge transferred in a quantum-classical transition, Ω is the circular frequency of the absorbed photon, $M_{\text{A}}$ is the Avogadro constant**,** c is the speed of light in vacuum, $n_{\text{ref}}$ is the refractive index, and the quantity $K=K\left(\omega_{12}\right)$ is

(94) $$K=Av\left(m_{\iota 1}\right)\sum_{...m_{\iota 2}...}{\left|A_{12}\right|}^{2}\Delta \left[\omega_{12}-\sum_{\iota }\omega_{\iota }\left(m_{\iota 2}-m_{\iota 1}\right)\right]\text{.}$$

Here, the dependence of the amplitude $A_{12}$ on all transient (tunneling and above-barrier) states $...m_{\iota }...$ is determined by Eq. (80) [11, 79], the external summation is performed over all final states $...m_{\iota 2}...$ on the surface

(95) $$\sum_{\iota }\omega_{\iota }\left(m_{\iota 2}-m_{\iota 1}\right)=\omega_{12}$$

in concordance with the law of energy conservation

(96) $$\hbar \Omega =J_{1}-J_{2}+\hbar \omega_{12}$$

($J_{1}$ and $J_{2}$ are the electron binding energies in the initial and final states 1 and 2, see also Section 2.4.1 above), $Av\left(m_{\iota 1}\right)$ is the average of the initial states $...m_{\iota 1}...$ over the distribution function of Planck. The wavelength λ (see Section 1 above, Figs. 1 and 2) refers to the frequency Ω in Eq. (96) by the formula $\lambda ={2\pi c/\Omega n}_{\text{ref}}$.

In Eq. (80), let us change the summing over the transient (intermediate) states $...m_{\iota }...$ in all modes ι by summing on the surface

(74a) $$\sum_{\iota }\omega_{\iota }\left(m_{\iota }-m_{\iota 1}\right)=\omega_{1}$$

(compare with Eq. (74) in Section 2.4.4) and by the sum over all integers $\omega_{1}$ from −∞ to +∞. Then, taking into account Eq. (89), we obtain the amplitude of electron-charge transfers $A_{12}$ in the following form:

(97) $$A_{12}=c_{12}\sum_{l}\omega_{l}{\tilde{p}}_{l1}\sum_{\omega_{1}=-\infty }^{\infty }G^{\text{E}}\left(\omega_{1},L\right)P_{l}\left(m_{\iota 2},m_{\iota 1};\omega_{1}\right)\text{,}$$

(98) $$P_{l}\left(m_{\iota 2},m_{\iota 1};\omega_{1}\right)\equiv \sum_{...m_{\iota }...}\frac{o\left(m_{l},m_{l1}\right)}{r\left(m_{l},m_{l1}\right)}\prod_{\iota }r\left(m_{\iota 2},m_{\iota }\right)r\left(m_{\iota },m_{\iota 1}\right)\Delta \left[\omega_{1}-\sum_{\iota }\omega_{\iota }\left(m_{\iota }-m_{\iota 1}\right)\right]\text{,}$$

where the Green's function of the elementary electron transfers $G^{\text{E}}\left(\omega_{1},L\right)$ is given by Eqs. (91), (92), and (78), and $P_{l}\left(m_{\iota 2},m_{\iota 1};\omega_{1}\right)$ denotes the advanced phonon factor, which, due to the completeness of the system of phonon wave functions $\Phi_{0...m_{\iota }...}\left(p\right)$ (see Eq. (79)), transforms into a Franck-Condon type factor

(99) $$P_{l}^{\text{FC}}\left(m_{\iota 2},m_{\iota 1}\right)=\frac{o\left(m_{l2},m_{l1}\right)}{r\left(m_{l2},m_{l1}\right)}\prod_{\iota }r\left(m_{\iota 2},m_{\iota 1}\right)\text{ ,}$$

if in the Green's function of elementary electron-charge transfers $G^{\text{E}}\left(\omega_{1},L\right)$ entering into Eq. (97), we put the number $\omega_{1}=\text{constant}$:

(100) $$A_{12}=c_{12}G^{\text{E}}\left(\omega_{1}=\text{constant},L\right)\sum_{l}\omega_{l}{\tilde{p}}_{l1}P_{l}^{\text{FC}}\left(m_{\iota 2},m_{\iota 1}\right)\text{.}$$

The factor $P_{l}\left(m_{\iota 2},m_{\iota 1};\omega_{1}\right)$ (Eq. (98)) take appropriately all possible virtual electron and nuclear movements into account, which create intermediate energy levels $J_{1}+\hbar \omega_{1}$ of the electron.

To calculate the factor $P_{l}\left(m_{\iota 2},m_{\iota 1};\omega_{1}\right)$ and other similar quantities further, we need the Cauchy theorem with respect to the Laurent series:

(101) $$f\left(w\right)=\sum_{m=-\infty }^{\infty }b_{m}{\left(w-w_{0}\right)}^{m},\;b_{m}=\frac{1}{2\pi \;i}\oint \frac{f\left(\zeta \right)}{{\left(\zeta -w_{0}\right)}^{m+1}}d\zeta \text{,}$$

where the contour encircles the point $w_{0}$, in the particular case $w_{0}=0$.

So, to calculate the factor $P_{l}\left(m_{\iota 2},m_{\iota 1};\omega_{1}\right)$ in Eq. (98), we introduce the generating polynomials

(102) $$rr\left(m_{\iota 2},m_{\iota 1};u\right)\equiv r\left(m_{\iota 2},m_{\iota 1}\right)r\left(m_{\iota 1},m_{\iota 1}\right)+u^{\omega_{\iota }}r\left(m_{\iota 2},m_{\iota 1}+1\right)r\left(m_{\iota 1}+1,m_{\iota 1}\right)+...+u^{-\omega_{\iota }}r\left(m_{\iota 2},m_{\iota 1}-1\right)r\left(m_{\iota 1}-1,m_{\iota 1}\right)+...$$

and

(103) $$ro\left(m_{l2},m_{l1};u\right)\equiv r\left(m_{l2},m_{l1}\right)o\left(m_{l1},m_{l1}\right)+u^{\omega_{l}}r\left(m_{l2},m_{l1}+1\right)o\left(m_{l1}+1,m_{l1}\right)+...+u^{-\omega_{l}}r\left(m_{l2},m_{l1}-1\right)o\left(m_{l1}-1,m_{l1}\right)+...\text{,}$$

where the Condon integrals r(...) and o(...) are given by Eqs. (82) and (83), correspondingly. Then, using the Cauchy theorem with respect to the Laurent series (see Eq. (101)), from Eq. (98) we obtain

(104) $$P_{l}\left(m_{\iota 2},m_{\iota 1};\omega_{1}\right)=\frac{1}{2\pi i}\oint \frac{du}{u^{\omega_{1}+1}}\frac{ro\left(m_{l2},m_{l1};u\right)}{rr\left(m_{l2},m_{l1};u\right)}\prod_{\iota }rr\left(m_{\iota 2},m_{\iota 1};u\right)\text{,}$$

where the contour encircles the point u=0. Substituting the amplitude $A_{12}$ from Eq. (97) into Eq. (94) for the optical absorption K, we have

(105) $$K=c_{12}^{2}\sum_{\omega_{1}=-\infty }^{\infty }G^{\text{E}}\left(\omega_{1},L\right)\sum_{\omega_{1}^{\prime }=-\infty }^{\infty }G^{\text{E}}*\left(\omega_{1}^{\prime },L\right)\sum_{l}\omega_{l}{\tilde{p}}_{l1}\sum_{l^{\prime }}\omega_{l^{\prime }}{\tilde{p}}_{l^{\prime }1}Av\left(m_{\iota 1}\right)\sum_{...m_{\iota 2}...}P_{l}\left(m_{\iota 2},m_{\iota 1};\omega_{1}\right)P_{l^{\prime }}\left(m_{\iota 2},m_{\iota 1};\omega_{1}^{\prime }\right)\Delta \left[\omega_{12}-\sum_{\iota }\omega_{\iota }\left(m_{\iota 2}-m_{\iota 1}\right)\right]\text{,}$$

where $P_{l^{\prime }}\left(m_{\iota 2},m_{\iota 1};\omega_{1}^{\prime }\right)$ is the advanced phonon factor arising from the complex conjugate amplitude $A_{12}*$. By the same method that we got the result (104), we get

(106) $$P_{l^{\prime }}\left(m_{\iota 2},m_{\iota 1};\omega_{1}^{\prime }\right)=\frac{1}{2\pi \;i}\oint \frac{dv}{v^{\omega_{1}^{\prime }+1}}\frac{ro\left(m_{l^{\prime }2},m_{l^{\prime }1};v\right)}{rr\left(m_{l^{\prime }2},m_{l^{\prime }1};v\right)}\prod_{\iota }rr\left(m_{\iota 2},m_{\iota 1};v\right)\text{.}$$

The quantity

(107) $$Av\left(m_{\iota 1}\right)\sum_{...m_{\iota 2}...}P_{l}\left(m_{\iota 2},m_{\iota 1};\omega_{1}\right)P_{l^{\prime }}\left(m_{\iota 2},m_{\iota 1};\omega_{1}^{\prime }\right)\Delta \left[\omega_{12}-\sum_{\iota }\omega_{\iota }\left(m_{\iota 2}-m_{\iota 1}\right)\right]$$

in Eq. (105) will be hereinafter referred to as $R_{l,l^{\prime }}\left(\omega_{1},\omega_{1}^{\prime };\omega_{12}\right)$. This function describes the electron-phonon dynamics for the individual levels $\omega_{1}$ and $\omega_{1}^{\prime }$ and the phonon modes l and $l^{\prime }$, which is due to the virtual electron-phonon coupling. Note that the quantity

(108) $$\sum_{l}\omega_{l}{\tilde{p}}_{l1}\sum_{l^{\prime }}\omega_{l^{\prime }}{\tilde{p}}_{l^{\prime }1}R_{l,l^{\prime }}\left(\omega_{1},\omega_{1}^{\prime };\omega_{12}\right)\equiv \phi \left(\omega_{1},\omega_{1}^{\prime };\omega_{12}\right)$$

(see also the result for the function $\phi \left(\omega_{1},\omega_{1}^{\prime };\omega_{12}\right)$ in Eq. (120) below) describes the electron-phonon dynamics for the individual levels $\omega_{1}$ and $\omega_{1}^{\prime }$, which is caused by the virtual electron-phonon coupling, taking into account all the phonon modes.

So, using the function $R_{l,l^{\prime }}\left(\omega_{1},\omega_{1}^{\prime };\omega_{12}\right)$, the formula (105) for the optical absorption K is rewritten as follows:

(109) $$K\equiv K\left(\omega_{12}\right)=c_{12}^{2}\sum_{\omega_{1}=-\infty }^{\infty }G^{\text{E}}\left(\omega_{1},L\right)\sum_{\omega_{1}^{\prime }=-\infty }^{\infty }G^{\text{E}}*\left(\omega_{1}^{\prime },L\right)\sum_{l}\omega_{l}{\tilde{p}}_{l1}\sum_{l^{\prime }}\omega_{l^{\prime }}{\tilde{p}}_{l^{\prime }1}R_{l,l^{\prime }}\left(\omega_{1},\omega_{1}^{\prime };\omega_{12}\right)$$

Substituting the factors $P_{l}\left(m_{\iota 2},m_{\iota 1};\omega_{1}\right)$ and $P_{l^{\prime }}\left(m_{\iota 2},m_{\iota 1};\omega_{1}^{\prime }\right)$, expressed in terms of contour integrals and polynomials (Eqs. (102) and (103)), from Eqs. (104) and (106) into the expression (107), we have

(110) $$R_{l,l^{\prime }}\left(\omega_{1},\omega_{1}^{\prime };\omega_{12}\right)=Av\left(m_{\iota 1}\right)\frac{1}{{\left(2\pi i\right)}^{2}}\oint \frac{du}{u^{\omega_{1}+1}}\oint \frac{dv}{v^{\omega_{1}^{\prime }+1}}\sum_{...m_{\iota 2}...}\Delta \left[\omega_{12}-\sum_{\iota }\omega_{\iota }\left(m_{\iota 2}-m_{\iota 1}\right)\right]\frac{ro\left(m_{l2},m_{l1};u\right)ro\left(m_{l^{\prime }2},m_{l^{\prime }1};v\right)}{rr\left(m_{l2},m_{l1};u\right)rr\left(m_{l^{\prime }2},m_{l^{\prime }1};v\right)}\prod_{\iota }rr\left(m_{\iota 2},m_{\iota 1};u\right)rr\left(m_{\iota 2},m_{\iota 1};v\right)\text{.}$$

Further, in Eq. (110) we perform summation over all final states 2, using, as in the derivation of Eq. (104), the Cauchy theorem with respect to the Laurent series (see Eq. (101)). For $l\neq l^{\prime }$ we get

(111) $$R_{l,l^{\prime }}\left(\omega_{1},\omega_{1}^{\prime };\omega_{12}\right)=Av\left(m_{\iota 1}\right)\frac{1}{{\left(2\pi i\right)}^{3}}\oint \frac{du}{u^{\omega_{1}+1}}\oint \frac{dv}{v^{\omega_{1}^{\prime }+1}}\oint \frac{dw}{w^{\omega_{12}+1}}rorr\left(m_{l1};u,v,w\right)rrro\left(m_{l^{\prime }1};u,v,w\right)\prod_{\iota }rrrr\left(m_{\iota 1};u,v,w\right)\text{,}$$

where the contours encircle the points u=0, v=0 and w=0, correspondingly; the generating polynomials entering here are determined in the following way:

(112) $$rrrr\left(m_{\iota 1};u,v,w\right)\equiv rr\left(m_{\iota 1},m_{\iota 1};u\right)rr\left(m_{\iota 1},m_{\iota 1};v\right)+w^{\omega_{\iota }}rr\left(m_{\iota 1}+1,m_{\iota 1};u\right)rr\left(m_{\iota 1}+1,m_{\iota 1};v\right)+...+w^{-\omega_{\iota }}rr\left(m_{\iota 1}-1,m_{\iota 1};u\right)rr\left(m_{\iota 1}-1,m_{\iota 1};v\right)+...\text{,}$$

(113) $$rorr\left(m_{l1};u,v,w\right)rrrr\left(m_{l1};u,v,w\right)\equiv ro\left(m_{l1},m_{l1};u\right)rr\left(m_{l1},m_{l1};v\right)+w^{\omega_{l}}ro\left(m_{l1}+1,m_{l1};u\right)rr\left(m_{l1}+1,m_{l1};v\right)+...+w^{-\omega_{l}}ro\left(m_{l1}-1,m_{l1};u\right)rr\left(m_{l1}-1,m_{l1};v\right)+...$$

and

(114) $$rrro\left(m_{l^{\prime }1};u,v,w\right)rrrr\left(m_{l^{\prime }1};u,v,w\right)\equiv rr\left(m_{l^{\prime }1},m_{l^{\prime }1};u\right)ro\left(m_{l^{\prime }1},m_{l^{\prime }1};v\right)+w^{\omega_{l^{\prime }}}rr\left(m_{l^{\prime }1}+1,m_{l^{\prime }1};u\right)ro\left(m_{l^{\prime }1}+1,m_{l^{\prime }1};v\right)+...+w^{-\omega_{l^{\prime }}}rr\left(m_{l^{\prime }1}-1,m_{l^{\prime }1};u\right)ro\left(m_{l^{\prime }1}-1,m_{l^{\prime }1};v\right)+...\text{.}$$

For $l=l^{\prime }$, the function $R_{l,l^{\prime }}\left(\omega_{1},\omega_{1}^{\prime };\omega_{12}\right)$ is formulated as

(115) $$R_{l,l}\left(\omega_{1},\omega_{1}^{\prime };\omega_{12}\right)=Av\left(m_{\iota 1}\right)\frac{1}{{\left(2\pi i\right)}^{3}}\oint \frac{du}{u^{\omega_{1}+1}}\oint \frac{dv}{v^{\omega_{1}^{\prime }+1}}\oint \frac{dw}{w^{\omega_{12}+1}}roro\left(m_{l1};u,v,w\right)\prod_{\iota }rrrr\left(m_{\iota 1};u,v,w\right)\text{,}$$

where the generating polynomials

(116) $$roro\left(m_{l1};u,v,w\right)rrrr\left(m_{l1};u,v,w\right)\equiv ro\left(m_{l1},m_{l1};u\right)ro\left(m_{l1},m_{l1};v\right)+w^{\omega_{l}}ro\left(m_{l1}+1,m_{l1};u\right)ro\left(m_{l1}+1,m_{l1};v\right)+...+w^{-\omega_{l}}ro\left(m_{l1}-1,m_{l1};u\right)ro\left(m_{l1}-1,m_{l1};v\right)+...\text{,}$$

and the generating polynomials $rrrr\left(m_{\iota 1};u,v,w\right)$ are provided by Eq. (112). The total electron-phonon dynamics due to the virtual electron-phonon coupling is taken into account by the sum over all transient (intermediate) levels of the electron energy $\hbar \omega_{1}$ (and the sum over all phonon modes l) in the optical absorption K. After the substitution of the functions $R_{l,l^{\prime }}\left(\omega_{1},\omega_{1}^{\prime };\omega_{12}\right)$ and $R_{l,l}\left(\omega_{1},\omega_{1}^{\prime };\omega_{12}\right)$ from Eqs. (111) and (115) into Eq. (109) for K, we obtain finally [11, 79].

(117) $$K=c_{12}^{2}\sum_{\omega_{1}=-\infty }^{\infty }G^{\text{E}}\left(\omega_{1},L\right)\sum_{\omega_{1}^{\prime }=-\infty }^{\infty }G^{\text{E}}*\left(\omega_{1}^{\prime },L\right)\frac{1}{{\left(2\pi i\right)}^{3}}\oint \frac{du}{u^{\omega_{1}+1}}\oint \frac{dv}{v^{\omega_{1}^{\prime }+1}}\oint \frac{dw}{w^{\omega_{12}+1}}Av\left(m_{1}\right)\left[P\left(m_{1};u,v,w\right)R\left(m_{1};u,v,w\right)\right]\text{,}$$

where the generating polynomial

(118) $$P\left(m_{1};u,v,w\right)\equiv \sum_{l}\sum_{l^{\prime }\neq l}\omega_{l}\omega_{l^{\prime }}{\tilde{p}}_{l1}{\tilde{p}}_{l^{\prime }1}rorr\left(m_{l1};u,v,w\right)rrro\left(m_{l^{\prime }1};u,v,w\right)+\sum_{l}\omega_{l}^{2}{\tilde{p}}_{l1}^{2}roro\left(m_{l1};u,v,w\right)$$

and the generating polynomial

(119) $$R\left(m_{1};u,v,w\right)\equiv \prod_{\iota }rrrr\left(m_{\iota 1};u,v,w\right)\text{;}$$

the generating polynomials $rrrr\left(m_{\iota 1};u,v,w\right)$, $rorr\left(m_{l1};u,v,w\right)$, $rrro\left(m_{l^{\prime }1};u,v,w\right)$ and $roro\left(m_{l1};u,v,w\right)$ are provided by Eqs. (112), (113), (114), and (116), respectively (see also the polynomials in Eqs. (102) and (103) above). Note that the result for the function $\phi \left(\omega_{1},\omega_{1}^{\prime };\omega_{12}\right)$, which describes the electron-phonon dynamics for the individual levels $\omega_{1}$ and $\omega_{1}^{\prime }$ because of the virtual electron-phonon coupling (with allowance for all phonon modes) and being determined above according to Eq. (108), is the following:

(120) $$\phi \left(\omega_{1},\omega_{1}^{\prime };\omega_{12}\right)\equiv \phi \left(\omega_{1},\omega_{1}^{\prime };T;\omega_{12}\right)=\frac{1}{{\left(2\pi i\right)}^{3}}\oint \frac{du}{u^{\omega_{1}+1}}\oint \frac{dv}{v^{\omega_{1}^{\prime }+1}}\oint \frac{dw}{w^{\omega_{12}+1}}Av\left(m_{1}\right)\left[P\left(m_{1};u,v,w\right)R\left(m_{1};u,v,w\right)\right]\text{,}$$

where T is the absolute temperature. Finally, in the resulting Eq. (117) for the optical absorption K, the Green's function of elementary electron transfers $G^{\text{E}}\left(\omega_{1},L\right)$ is given by Eqs. (91), (92), and (78), and the constant $c_{12}$ is given by Eq. (90).

#### 2.4.7 The case of non-local phonons

For simplicity, we do not take into account local vibrations, and so we consider below the average of the initial states $...m_{1}...$ over the distribution function of Planck,

(121) $$Av\left(m_{1}\right)\left[P\left(m_{1};u,v,w\right)R\left(m_{1};u,v,w\right)\right]$$

(see this term in Eq. (117)), merely for non-local vibrations of nuclei (crystal lattice phonons). In this case, we can take advantage of the fact that

(122) $${\tilde{p}}_{\iota }∼M^{-1/2},$$

where M is the amount of multipliers in the generating polynomial $P\left(m_{1};u,v,w\right)R\left(m_{1};u,v,w\right)$ (see Eqs. (118) and (119)), which is equal to the amount of vibrational degrees of freedom in a condensed medium. Being mindful of the limiting transition M→∞ in the final result, it suffices to hold terms in the polynomial $P\left(m_{1};u,v,w\right)R\left(m_{1};u,v,w\right)$ pending order $M^{-1}$ inclusively. The Condon integrals in Eqs. (82) and (83) are easily computed by expanding in powers of ${\tilde{p}}_{\iota }$, and we obtain

$$r\left(m_{\iota 1},m_{\iota 1}\right)=1-\frac{{\tilde{p}}_{\iota }^{2}}{2}\left(m_{\iota 1}+\frac{1}{2}\right)+...\text{,}\;r\left(m_{\iota 1}+1,m_{\iota 1}\right)={\tilde{p}}_{\iota }\sqrt{\frac{m_{\iota 1}+1}{2}}+...\text{,}$$

(123) $$r\left(m_{\iota 1}-1,m_{\iota 1}\right)=-{\tilde{p}}_{\iota }\sqrt{\frac{m_{\iota 1}}{2}}+...\;\text{;}$$

$$o\left(m_{l1},m_{l1}\right)=\frac{{\tilde{p}}_{l}}{2}+...\text{,}\;o\left(m_{l1}+1,m_{l1}\right)=\sqrt{\frac{m_{l1}+1}{2}}+...\text{,}$$

(124) $$o\left(m_{l1}-1,m_{l1}\right)=\sqrt{\frac{m_{l1}}{2}}+...\text{ .}$$

Considering Eqs. (122), (123), and (124), it is readily seen that transitions at which one of the phonon occupation numbers changes by over 1 do not contribute to the polynomial $P\left(m_{1};u,v,w\right)R\left(m_{1};u,v,w\right)$. Substituting the results (123) and (124) into the polynomials (102) and (103) and then substituting the polynomials (102) and (103) into the polynomials (112, 113, 114) and (116), we have the following results for generating polynomials of three variables:

(125) $$rrrr\left(m_{\iota 1};u,v,w\right)=1-\left({\tilde{p}}_{\iota 1}^{2}+{\tilde{p}}_{\iota 2}^{2}\right)\left(m_{\iota 1}+\frac{1}{2}\right)+\frac{1}{2}{\tilde{p}}_{\iota 1}^{2}\left[\left(m_{\iota 1}+1\right){\left(uvw\right)}^{\omega_{\iota }}+m_{\iota 1}{\left(uvw\right)}^{-\omega_{\iota }}\right]+\frac{1}{2}{\tilde{p}}_{\iota 2}^{2}\left[\left(m_{\iota 1}+1\right)w^{\omega_{\iota }}+m_{\iota 1}w^{-\omega_{\iota }}\right]-\frac{1}{2}{\tilde{p}}_{\iota 1}{\tilde{p}}_{\iota 2}\left[\left(m_{\iota 1}+1\right)\left(u^{\omega_{\iota }}+v^{\omega_{\iota }}\right)\left(w^{\omega_{\iota }}-1\right)+m_{\iota 1}\left(u^{-\omega_{\iota }}+v^{-\omega_{\iota }}\right)\left(w^{-\omega_{\iota }}-1\right)\right]\text{,}$$

(126) $$rorr\left(m_{l1};u,v,w\right)rrrr\left(m_{l1};u,v,w\right)=\frac{{\tilde{p}}_{l1}}{2}\left[1+\left(m_{l1}+1\right){\left(uvw\right)}^{\omega_{l}}-m_{l1}{\left(uvw\right)}^{-\omega_{l}}\right]-\frac{{\tilde{p}}_{l2}}{2}\left[\left(m_{l1}+1\right)u^{\omega_{l}}\left(w^{\omega_{l}}-1\right)-m_{l1}u^{-\omega_{l}}\left(w^{-\omega_{l}}-1\right)\right]\text{,}$$

(127) $$rrro\left(m_{l1};u,v,w\right)rrrr\left(m_{l1};u,v,w\right)=\frac{{\tilde{p}}_{l1}}{2}\left[1+\left(m_{l1}+1\right){\left(vuw\right)}^{\omega_{l}}-m_{l1}{\left(vuw\right)}^{-\omega_{l}}\right]-\frac{{\tilde{p}}_{l2}}{2}\left[\left(m_{l1}+1\right)v^{\omega_{l}}\left(w^{\omega_{l}}-1\right)-m_{l1}v^{-\omega_{l}}\left(w^{-\omega_{l}}-1\right)\right]=rorr\left(m_{l1};v,u,w\right)rrrr\left(m_{l1};v,u,w\right)=rorr\left(m_{l1};v,u,w\right)rrrr\left(m_{l1};u,v,w\right)\text{,}$$

(128) $$roro\left(m_{l1};v,u,w\right)rrrr\left(m_{l1};u,v,w\right)=\frac{1}{2}\left[\left(m_{l1}+1\right){\left(uvw\right)}^{\omega_{l}}+m_{l1}{\left(uvw\right)}^{-\omega_{l}}\right]\text{.}$$

By virtue of the relation (122) and in accordance with Eqs. (125), (126), (127), and (128), the generating polynomial $P\left(m_{1};u,v,w\right)R\left(m_{1};u,v,w\right)$ (Eqs. (117, 118, 119)) can be formulated as the product of M→∞ statistically independent multipliers that are linearly dependent on $m_{1}$. Consequently, the operation of averaging $P\left(m_{1};u,v,w\right)R\left(m_{1};u,v,w\right)$ is reduced to replacement of all $m_{\iota 1}$ and $m_{l1}$ by their average equilibrium values ${\bar{m}}_{\iota 1,l1}$ (Planck's distribution function):

(129) $${\bar{m}}_{\iota 1,l1}={\left[\exp\left(\hbar \omega_{\iota ,l}/k_{\text{B}}T\right)-1\right]}^{-1}\text{.}$$

As a result, the generating functions $R\left({\bar{m}}_{1};u,v,w\right)$ and $P\left({\bar{m}}_{1};u,v,w\right)$ and optical absorption K [11, 79] are expressed as follows:

(130) $$K=c_{12}^{2}\sum_{\omega_{1}=-\infty }^{\infty }\sum_{\omega_{1}^{\prime }=-\infty }^{\infty }G^{\text{E}}\left(\omega_{1},L\right)G^{\text{E}}*\left(\omega_{1}^{\prime },L\right)\frac{1}{{\left(2\pi \;i\right)}^{3}}\oint \frac{du}{u^{\omega_{1}+1}}\oint \frac{dv}{v^{\omega_{1}^{\prime }+1}}\oint \frac{dw}{w^{\omega_{12}+1}}P\left({\bar{m}}_{1};u,v,w\right)R\left({\bar{m}}_{1};u,v,w\right)\text{,}$$

(131) $$R\left({\bar{m}}_{1};u,v,w\right)=\exp\{-\sum_{\iota }{\tilde{p}}_{\iota }^{2}\left(2{\bar{m}}_{\iota 1}+1\right)+\frac{1}{2}\sum_{\iota }{\tilde{p}}_{\iota }^{2}[\left({\bar{m}}_{\iota 1}+1\right)\left(u^{\omega_{\iota }}v^{\omega_{\iota }}+1\right)w^{\omega_{\iota }}+{\bar{m}}_{\iota 1}\left(u^{-\omega_{\iota }}v^{-\omega_{\iota }}+1\right)w^{-\omega_{\iota }}]\}\text{,}$$

(132) $$P\left({\bar{m}}_{1};u,v,w\right)=\frac{1}{4}{\left\{\sum_{l}\omega_{l}{\tilde{p}}_{l}^{2}\left[1+\left({\bar{m}}_{l1}+1\right){\left(uvw\right)}^{\omega_{l}}-{\bar{m}}_{l1}{\left(uvw\right)}^{-\omega_{l}}\right]\right\}}^{2}+\frac{1}{2}\sum_{l}\omega_{l}^{2}{\tilde{p}}_{l}^{2}\left[\left({\bar{m}}_{l1}+1\right){\left(uvw\right)}^{\omega_{l}}+{\bar{m}}_{l1}{\left(uvw\right)}^{-\omega_{l}}\right]\text{.}$$

We note that for simplicity, the small terms ${\tilde{p}}_{\iota 1}{\tilde{p}}_{\iota 2}\propto \frac{1}{L}$ and ${\tilde{p}}_{l1}{\tilde{p}}_{l2}\propto \frac{1}{L}$, related to the phonon correlations, are neglected in the generating functions $R\left({\bar{m}}_{1};u,v,w\right)$ and $P\left({\bar{m}}_{1};u,v,w\right)$.

#### 2.4.8 The analytical result for the shape of the optical absorption band

In the expression (130, 131, 132) for the optical absorption K, we expand the logarithm of the Green's function of electron transfers $G^{\text{E}}\left(\omega_{1},L\right)\equiv G^{\text{E}}\left(J_{1}+\hbar \omega_{1};L\right)$ (see Eqs. (91) and (92) in Section 2.4.5) with respect to the intermediate levels of the electron energy $\hbar \omega_{1}$ in the vicinity of the donor level $J_{1}$. Restricting ourselves to the linear approximation, we have

(133) $$G^{\text{E}}\left(J_{1}+\hbar \omega_{1};L\right)\approx G^{\text{E}}\left(J_{1};L\right)\exp\left(-t\omega_{1}\right)\text{,}$$

(134) $$t=-{\left[\partial \ln G^{\text{E}}\left(J_{1}+\hbar \omega_{1};L\right)/\partial \omega_{1}\right]}_{\omega_{1}=0}\text{.}$$

Substituting the approximate expression (133) into the exact result (130, 131, 132) and applying the Cauchy theorem with respect to the Laurent series (see Eq. (101)), we get [11, 79].

(135) $$K=c_{12}^{2}{\left|G^{\text{E}}\left(J_{1};L\right)\right|}^{2}\frac{1}{2\pi i}\oint \frac{dw}{w^{\omega_{12}+1}}P\left[{\bar{m}}_{1};u=\exp\left(-t\right),v=\exp\left(-t\right),w\right]R\left[{\bar{m}}_{1};u=\exp\left(-t\right),v=\exp\left(-t\right),w\right]\text{.}$$

It goes without saying that the application of the linear approximation to $\ln\left[G^{\text{E}}\left(J_{1}+\hbar \omega_{1};L\right)\right]$ (Eq. (133)) imposes restrictions on the problem parameters; however, it can be shown that these restrictions are not severe [52].

Methods for calculating the contour integral in Eq. (135) are similar to those applied in the standard theory of many-phonon transitions (Ref. [72]). In Eq. (135), the generating function $R\left[{\bar{m}}_{1};u=\exp\left(-t\right),v=\exp\left(-t\right),w\right]$ (Eq. (131)) is exponential with respect to the variable w, while the generating function $P\left[{\bar{m}}_{1};u=\exp\left(-t\right),v=\exp\left(-t\right),w\right]$ (Eq. (132)) is a power function in w. Consequently, in a greater in modulus thermal effect $\hbar \omega_{12}$, when the phonon frequencies

(136) $$\omega_{\iota ,l}<<\left|\omega_{12}\right|\text{,}$$

we can put $\omega_{l}$ equal to zero in quantity $w^{\pm \omega_{l}}$, which appears in $P\left[{\bar{m}}_{1};u=\exp\left(-t\right),v=\exp\left(-t\right),w\right]$.^15^ Thus, P is not now dependent on w, and in Eq. (135) it can be brought outside the integral character in the point w=1 [11, 79]:

(137) $$K=c_{12}^{2}{\left|G^{\text{E}}\left(J_{1};L\right)\right|}^{2}P\left[{\bar{m}}_{1};u=\exp\left(-t\right),v=\exp\left(-t\right),w=1\right]\frac{1}{2\pi i}\oint \frac{dw}{w^{\omega_{12}+1}}R\left[{\bar{m}}_{1};u=\exp\left(-t\right),v=\exp\left(-t\right),w\right]\text{.}$$

Within the framework of Einstein's model of the vibrations of nuclei $\omega_{\iota }=\text{constant}\equiv \omega$, in Eq. (137) the contour integral is calculated exactly. In order to obtain the result, one can use, for example, the well-known expansion in a series [85]:

(138) $$\exp\left(Az+\frac{B}{z}\right)=\sum_{n=-\infty }^{\infty }z^{n}{\left(\frac{A}{B}\right)}^{\frac{n}{2}}I_{n}\left(2\sqrt{AB}\right)\text{,}$$

where $I_{n}\left(...\right)$ is the modified Bessel function. Applying once again the Cauchy theorem with respect to the Laurent series (see Eq. (101)), from Eq. (137), taking Eq. (138) into account, we get

(139) $$K=c_{12}^{2}{\left|G^{\text{E}}\left(J_{1};L\right)\right|}^{2}P\left[{\bar{m}}_{1};u=\exp\left(-t\right),v=\exp\left(-t\right),w=1\right]\exp\left[-\frac{2E}{\hbar \omega }\text{cth }\beta_{T}+\left(\;\beta_{T}-t\right)\frac{\omega_{12}}{\omega }\right]\;I_{\frac{\omega_{12}}{\omega }}\left(\frac{2E\text{ ch }t}{\hbar \omega \text{ sh }\beta_{T}}\right),$$

where $\beta_{T}\equiv \hbar \omega /2k_{\text{B}}T$. Further, using the well-known asymptotic formula [85, 86]

(140) $$I_{\kappa }\left(u\right)\approx \frac{1}{\sqrt{2\pi u}}\exp\left(u-\frac{\kappa^{2}}{2u}\right),\;u>>1,\;\kappa \leq u\text{,}$$

as a result, we have optical absorption K, which is entirely expressed in elementary functions [10, 11, 12, 79]:

(141) $$K=K_{0}\;\exp\left(Y\right)\text{,}$$

(142) $$Y=\frac{1}{2}\ln\left(\frac{\omega \tau \text{ sinh }\beta_{T}}{4\pi \text{ cosh }t}\right)-\frac{2}{\omega \tau }\left(\text{coth }\beta_{T}-\frac{\text{cosh }t}{\text{sinh }\beta_{T}}\right)+\left(\beta_{T}-t\right)\frac{1}{\omega \tau \;\Theta }-\frac{\text{sinh }\beta_{T}}{4\omega \tau \;\Theta^{2}\text{cosh }t}\text{,}$$

(143) $$1<<\frac{1}{\omega \tau \;\Theta }\leq \frac{2\text{cosh }t}{\omega \tau \text{ sinh }\beta_{T}}\text{,}$$

where

(144) $$t=\frac{\omega \tau_{\text{e}}}{\theta }\left[\frac{AC+BD}{A^{2}+B^{2}}+\frac{2\Theta \left(\Theta -1\right)}{{\left(\Theta -1\right)}^{2}+{\left(\Theta /\theta_{0}\right)}^{2}}+\frac{{\theta_{0}}^{2}}{{\theta_{0}}^{2}+1}\right]\text{,}$$

(145) $$\left|\theta_{0}\right|>>\frac{E}{2J_{1}}\text{,}$$

(146) $$\theta \equiv \frac{\tau_{\text{e}}}{\tau }=\frac{L\;E}{\hbar \sqrt{2J_{1}/m}}\text{,}\;\Theta \equiv \frac{\tau^{\prime }}{\tau }=\frac{E}{\Delta },\;\theta_{0}\equiv \frac{\tau_{0}}{\tau }=\frac{E}{\gamma }\text{,}$$

(147) $$\tau_{\text{e}}=\frac{L}{\sqrt{2J_{1}/m}},\;\tau =\frac{\hbar }{E},\;\tau^{\prime }=\frac{\hbar }{\Delta },\;\tau_{0}=\frac{\hbar }{\gamma }\text{.}$$

Here, we use the notation

(148) $$A=\cos\left(\frac{\theta }{\theta_{0}}\right)+\Lambda +{\left(\frac{1}{\theta_{0}}\right)}^{2}\nu ,\;B=\sin\left(\frac{\theta }{\theta_{0}}\right)+\frac{1}{\theta_{0}}\mu \text{,}$$

(149) $$C=\theta \;\left[\cos\left(\frac{\theta }{\theta_{0}}\right)-\frac{1-\xi^{2}}{2\theta_{0}}\sin\left(\frac{\theta }{\theta_{0}}\right)\right]+\mu \text{,}$$

(150) $$D=\theta \;\left[\sin\left(\frac{\theta }{\theta_{0}}\right)+\frac{1-\xi^{2}}{2\theta_{0}}\cos\left(\frac{\theta }{\theta_{0}}\right)\right]-\frac{2}{\theta_{0}}\nu \text{,}$$

(151) $$\xi \equiv {\left(1-\frac{E}{J_{1}}\right)}^{1/2}\;\left(J_{1}>E\;\text{by definition}\right)\text{,}$$

and where we finally have

(152) $$\Lambda =-{\left(\Theta -1\right)}^{2}\varepsilon +\left[\frac{\left(\Theta -1\right)\theta }{\rho }+\Theta \left(\Theta -2\right)\right]\varepsilon^{\frac{1-\rho }{1-\xi }}\text{,}$$

(153) $$\mu =2\Theta \left(\Theta -1\right)\varepsilon -\left[\frac{\left(2\Theta -1\right)\theta }{\rho }+2\Theta \left(\Theta -1\right)\right]\varepsilon^{\frac{1-\rho }{1-\xi }}\text{,}$$

(154) $$\nu =\Theta \left[\Theta \varepsilon -\left(\frac{\theta }{\rho }+\Theta \right)\varepsilon^{\frac{1-\rho }{1-\xi }}\right]\text{,}$$

(155) $$\varepsilon \equiv \exp\left(\frac{2\theta }{1+\xi }\right),\;\rho \equiv \sqrt{\xi^{\;2}+\frac{1-\xi^{\;2}}{\Theta }}\text{.}$$

The factor $K_{0}$ becomes

(156) $$K_{0}=K_{0}^{e}K_{0}^{p}\text{,}$$

where

(157) $$K_{0}^{e}=\frac{2\tau^{3}J_{1}}{m}\frac{\left(A^{2}+B^{2}\right)\rho^{3}\Theta^{4}\xi }{\theta^{2}{\left[{\left(\Theta -1\right)}^{2}+{\left(\frac{\Theta }{\theta_{0}}\right)}^{2}\right]}^{2}\left[1+{\left(\frac{1}{\theta_{0}}\right)}^{2}\right]}\exp\left(-\frac{4\theta }{1-\xi^{2}}\right)$$

and

(158) $$K_{0}^{p}=\frac{1}{\omega \tau }{\left[1+\frac{\text{sinh}\left(\beta_{T}-2t\right)}{\text{sinh }\beta_{T}}\right]}^{2}+\frac{\text{cosh}\left(\beta_{T}-2t\right)}{\text{sinh }\beta_{T}}\text{.}$$

Conditions (143) and (145) are not any significant restrictions on the characteristics of the system and associated with points of regular approximations done in the computations [11, 79]. Inequalities (143) follow from conditions in formula (140). Inequality (145) follows from the expansion of the radical in a series by smallness of $\left|\gamma \right|/J_{1}$ in the Gamow exponent (see Section 2.4.4, formula (78) for $\omega_{1}=0$), which is limited to the zero-order approximation. The scaling times offered by Eq. (147) regulate the chaotic dynamics of quantum-classical transitions. We consider them at length in another place [12, 13, 14, 28, 79]. Here, they are discussed in a few words.

A crucial component in the dynamics of quantum-classical transitions is a numerical correlation between the time

(159) $$\tau_{\text{e}}=\frac{L}{\sqrt{2J_{1}/m}}$$

and the time

(160) $$\tau =\frac{\hbar }{E}\text{,}$$

that are elements in Eqs. (141), (142), (143), (144), (145), (146), (147), (148), (149), (150), (151), (152), (153), (154), (155), (156), (157), and (158) for computing optical absorption spectra (Eq. (147)). The time $\tau_{\text{e}}$ is the specific time of an electron movement between the donor and the acceptor, set apart by distance L. The time τ is the specific time of the environmental nuclear reorganization. In the case

(161) $${\left(2\tau_{e}\right)}^{-1}=\tau^{-1}$$

the so-called transferon resonance [12, 13] occurs between the respective frequencies ${\left(2\tau_{\text{e}}\right)}^{-1}$ and $\tau^{-1}$. We considered one of the implications of the resonance under discussion as an example in Ref. [18], Section 2.4 (optical absorption spectra as solution-dependent [17, 44]). Other its impacts are considered in Refs. [10, 11, 12, 14, 17, 28]. Farther, in Eq. (147), the time $\tau^{\prime }=\frac{\hbar }{\Delta }$ is the specific time of transformation of the energy ℏΩ of light into the excitation energy $J_{1}-J_{2}$ of electron and the heat energy $\Delta \equiv \hbar \omega_{12}$ in elementary electron-transfer processes (see Eq. (96)), and the time $\tau_{0}=\frac{\hbar }{\gamma }$ is the specific time of transformation of a movement (energy) of electron into a movement (energy) of nuclear reorganization (γ>0) and/or of the reverse acts (γ<0) in the transient dozy-chaos state (the sign of γ is discussed below). The non-dimensional parameters θ, Θ, $\theta_{0}$ (Eq. (146)) are related to the aforementioned specific times $\tau_{\text{e}}$, $\tau^{\prime }$, $\tau_{0}$, which are divided by the specific time τ (Eq. (160)). The quantities Λ=Λ(θ,Θ), μ=μ(θ,Θ) and ν=ν(θ,Θ) (see Eqs. (152), (153), and (154)), being independent of the parameter $\theta_{0}$ and the dozy-chaos energy γ, do not involve the chaos of the electron-nuclear movements in the transient state and describe merely the regular motion of the electron and nuclei. The functions $A=A\left(\theta ,\Theta ,\theta_{0}\right)$, $B=B\left(\theta ,\Theta ,\theta_{0}\right)$, $C=C\left(\theta ,\Theta ,\theta_{0}\right)$ and $D=D\left(\theta ,\Theta ,\theta_{0}\right)$ (see Eqs. (148), (149), and (150)), being dependent on the parameter $\theta_{0}$, already involve the chaos of the electron-nuclear movements in the transient state. The Eqs. (144), (145), (146), (147), (148), (149), (150), (151), (152), (153), (154), (155), and (157) are independent of the parameter $\beta_{T}\equiv \hbar \omega /2k_{\text{B}}T$ and the absolute temperature T, therefore, they do not include the averaging over the equilibrium distribution of the initial states in the ensemble of the donor-acceptor systems. By contrast, the Eqs. (142) and (158), being dependent on the parameter $\beta_{T}\equiv \hbar \omega /2k_{\text{B}}T$, already include the averaging over the equilibrium distribution of the initial states. All donor-acceptor systems in the ensemble have their own values of the parameter Θ, the corresponding thermal effect Δ and absorption frequency Ω of light (see Eq. (96)). These values, together with the other parameters of a donor-acceptor system in the ensemble, determine the position, width, intensity and shape of optical absorption bands. From the formula $\tau^{\prime }=\frac{\hbar }{\Delta }$ for the specific time of transformation of the light energy ℏΩ (see Eq. (147)) and from the law of energy conservation (see Eq. (96)) it follows that the dynamics of producing the shape of optical bands is fastest in the high-frequency tail of the optical bands and is slowest in their low-frequency tail. Lastly, in Eq. (157), $\exp\left[-4\theta /\left(1-\xi^{2}\right)\right]\equiv \exp\left(-2L/a\right)$ is the Gamow tunnel factor ($a\equiv \hbar /\sqrt{2mJ_{1}}$).

The quantity $K=K\left(\Theta ,\theta_{0}\right)$ (Eq. (141)) and the corresponding optical extinction (Eq. (93)) have a singularity at the point (Δ=E, γ=0) or $\left(\Theta =1,\;\theta_{0}=\infty \right)$. The nature of this singularity is controlled by singularities of the functions $t=t\left(\Theta ,\theta_{0}\right)$ in Eq. (144) and $K_{0}^{e}=K_{0}^{e}\left(\Theta ,\theta_{0}\right)$ in Eq. (157). The singularity in the function $K_{0}^{e}=K_{0}^{e}\left(\Theta ,\theta_{0}\right)$ is removable:

(162) $$\frac{K_{0}^{e}\left(\Theta =1,\;\theta_{0}\to \infty \right)}{2\tau^{3}J_{1}/m}=\frac{\xi }{\theta^{2}}{\left[\exp\left(\frac{2\theta }{1+\xi }\right)-\frac{\theta^{2}}{2}-\theta -1\right]}^{2}\;\exp\left(-\frac{4\theta }{1-\xi^{2}}\right)\text{.}$$

**Fig. 3:**
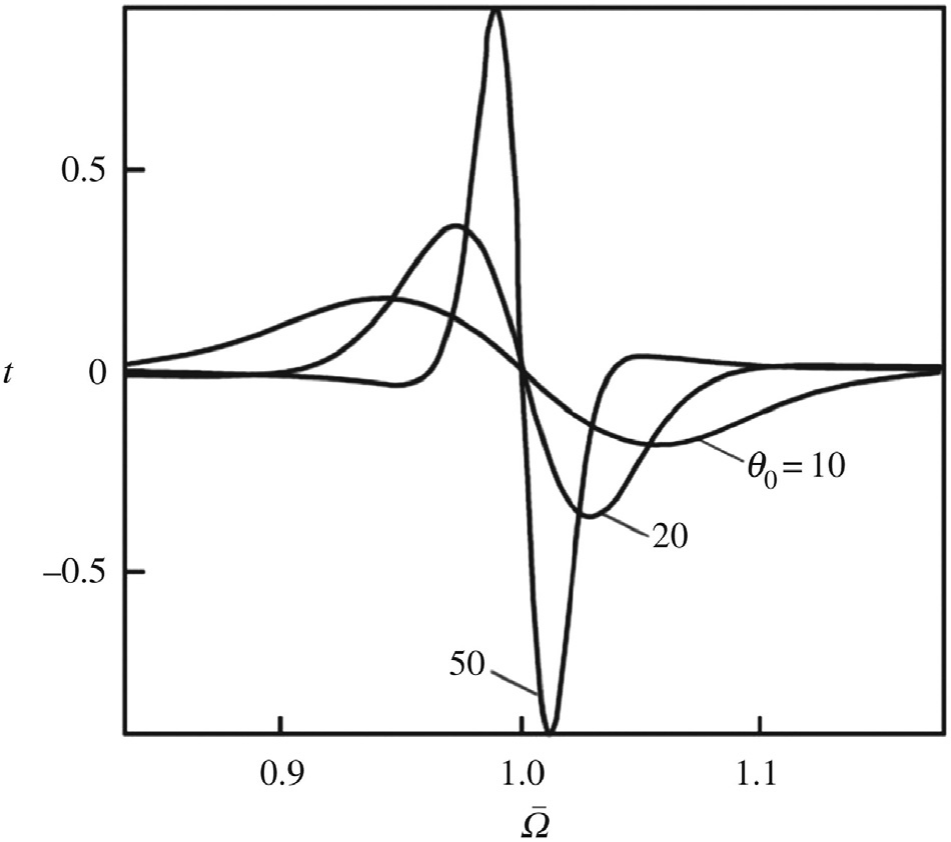
The dynamics of quantum-classical transitions in the neighborhood of the singular point $\left(\bar{\Omega }=1,\text{ ​}\theta_{0}=\infty \right)$ [(Δ=E, ​γ=0)] is demonstrated by the behaviour of the quantity $t=t\left(\bar{\Omega },\theta_{0}\right)$ (see Eq. (144)) [11]. For simplification, it is formally assumed here that $J_{2}-J_{1}=0$. Then $\Theta^{-1}=\hbar \Omega /E\equiv \bar{\Omega }$. The following system parameters are used: $J_{1}=5$ eV, E=1 eV, $\mu =m_{\text{e}}$, $\omega =5\times 10^{13}{\text{ ​s}}^{-1}$ and L=L∗≈0.44 nm (transferon resonance, Eq. (161)).

In the function $t=t\left(\Theta ,\theta_{0}\right)$, the singularity at the point $\left(\Theta =1,\;\theta_{0}=\infty \right)$ is irremovable. The behavior of the function $t=t\left(\Theta ,\theta_{0}\right)$ in the neighborhood of $\left(\bar{\Omega }\equiv \Theta^{-1}=1,\;\theta_{0}=\infty \right)$ is shown in Fig. 3. Must be noted that the result (141, 142, 143, 144, 145, 146, 147, 148, 149, 150, 151, 152, 153, 154, 155, 156, 157, 158) is invariable if you change the sign of γ. The invariancy is consistent with the physical case that both the virtual acts of transformation of electron movement (energy) into nuclear reorganization movement (energy) and the reverse acts occure in the transient dozy-chaos state [14, 15, 18, 28, 79]. For definiteness, we set γ>0 in Fig. 3 and hereinafter.

#### 2.4.9 Limit to standard result

**Fig. 4:**
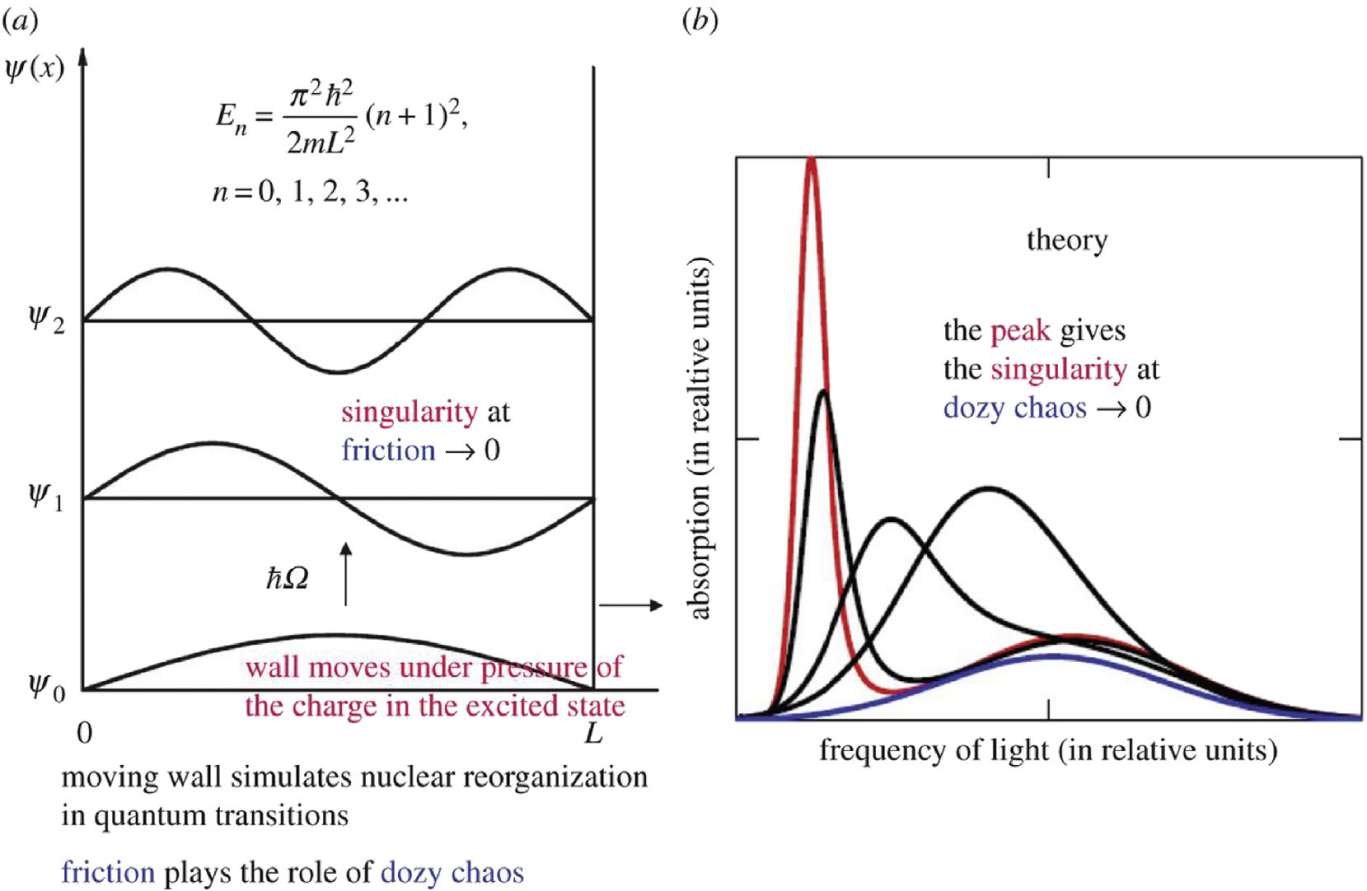
Singularity in the rate of molecular “quantum” transitions: a potential box with a moveable wall (*a*) and the optical absorption band shape dependant on the dozy chaos accessible to a certain “quantum” transition (*b*); the pronounced peak (J-band) matches the least dozy chaos [15, 18]. (Original citation) — Reproduced by permission of The Royal Society of Chemistry.

The limiting transition from the optical absorption K given by Eqs. (141), (142), (143), (144), (145), (146), (147), (148), (149), (150), (151), (152), (153), (154), (155), (156), (157), (158), and (96) to the corresponding result given by the standard many-phonon theory [72] can be *a priory* implemented by tending the dozy-chaos energy γ to either zero or infinity,^16^ but the quantity K turns out to be infinity for γ→0 and zero for γ→∞. The physical meaning of K(γ→0)→∞ beyond the adiabatic approximation is interpreted as a consequence of the incomparability of masses of the electron and its environmental nuclei (see Section 2.4.3). The physical meaning of K(γ→∞)→0 (see Fig. 4 in Section 2.6 below) is determined by the failure of electron transitions coupled to nuclear reorganizations when in the transient state the motion of nuclei is absolutely chaotic (random), hat is, when in the electron–nuclear system the internal friction is infinite. Given this, we can remove γ from Eqs. (141), (142), (143), (144), (145), (146), (147), (148), (149), (150), (151), (152), (153), (154), (155), (156), (157), (158), and (96) and get the standard result by tending γ to infinity in the equation for t (t→0; Fig. 3, where $\theta_{0}=E/\gamma$ under Eq. (146)) and to zero in $K_{0}^{e}$ (Eq. (162)). The standard type equation for K ($k_{\text{B}}T>\hbar \omega /2$) is thus obtained [11]:

(163) $$K=\frac{a^{2}\hbar }{\sqrt{4\pi \lambda_{\text{r}}k_{\text{B}}T}}\exp\left(-\frac{2L}{a}\right)\exp\left[-\frac{{\left(\Delta -\lambda_{\text{r}}\right)}^{2}}{4\lambda_{\text{r}}k_{\text{B}}T}\right]\text{,}$$

where $\lambda_{\text{r}}\equiv 2E$. A formula of this type was obtained by Marcus in his electron-transfer model [87, 88, 89, 90, 91, 92] and is often called the Marcus formula, and the energy $\lambda_{\text{r}}$ is called the reorganization energy of Marcus. Similar and more general formulas were previously obtained in the theory of many-phonon transitions (see Refs. [65, 72]) for optical transitions by Huang and Rhys [93] and Pekar [73, 74, 86] (see also Lax [94] and Krivoglaz and Pekar [76]) and for nonradiative transitions by Huang and Rhys [93] and Krivoglaz [77].

#### 2.4.10 Quantum-classical mechanics as applied to both solids and liquids

Although the basic principles of the quantum-classical mechanics of elementary electron transfers were given in this article in terms corresponding to solids, nowhere was the regular periodic configuration of atoms (nuclei) assumed. It was assumed only that the atoms make harmonic oscillations around some still equilibrium states. But such a condition also occurs in liquids for quite long periods of time until shaking happens due to a strong statistical fluctuation of the oscillations and new equilibrium states of the atoms are set up. Since the shaking time $\tau_{T}≅\hbar /k_{\text{B}}T$ greatly exceeds the elementary electron transfer time τ≅trτ=ℏ/E (Section 2.4.8, Eq. (160)), because of the irresistible condition $k_{\text{B}}T<<E$ (at room temperature $k_{\text{B}}T=1/40\text{ eV}$ and E=(1÷0.1) eV), our theory, such as the standard theory of many-phonon transitions [72, 73, 74], can be applied to both solids and liquids [11]. A clear confirmation of this is, for example, our successful theoretical explanations for the optical spectra in polymethine dyes and their aggregates in liquid solutions (Section 1).

### 2.5 Internal inconsistency of the Franck-Condon principle

As mentioned in Section 2.1, just as in quantum-classical mechanics, the transient state in the standard Franck-Condon picture is essentially classical: the classical motion of the nuclei is carried out to the turning point, or in other words, “towards the quantum transition.” In the standard theory, this qualitative Franck-Condon picture of the dynamics of the transient state does not have a formalized description. When we attempt to formalize this picture within the framework of quantum mechanics, we immediately obtain a singularity in the rates of molecular quantum transitions. This is because we use only quantum mechanics to describe molecular “quantum” transitions, whereas in the qualitative Franck-Condon picture, in addition to the quantum transition, we also have the classical motion of the nuclei “towards it.” Therefore, to eliminate the singularity, a certain paradigm of classicality must be introduced, in one way or another, into quantum mechanics. The simplest way to do this in the formal apparatus of quantum mechanics is to replace the infinitesimal imaginary additive in the energy denominator of the spectral representation of the total Green's function of the system by its finite value and thus come to quantum-classical mechanics. In the aforementioned reasoning, we did not have to resort, as we did earlier in the text of the article, to the argument that the mass of the nuclei is incomparably larger than the mass of the electrons (in this regard, see footnote 10 in Section 2.4.3). It was sufficient to refer to the internal inconsistency of the Franck-Condon picture, which is related with the fact that in theory, the qualitative Franck-Condon picture of the dynamics of the transient state does not have an adequate mathematical formulation.^17^

We previously discussed the internal inconsistency of the Franck-Condon principle related to the inability to formalize, within the framework of quantum mechanics, the qualitative quantum-classical picture of optical transitions in molecules, which the Franck-Condon principle offers. A similar internal inconsistency is inherent in the Franck-Condon principle when it is used in the standard theory of non-radiative elementary electron transfers in condensed media, namely, in Marcus' theory. In the qualitative picture of Marcus, it is assumed that the electron energy on the donor and on the acceptor in each individual donor-acceptor pair is equalized due to fluctuations in the classical motion of the nuclear subsystem (due to thermal fluctuations of the nuclear coordinates) before the quantum transition (transfer) of an electron occurs from the donor to the acceptor [91, 92]. In other words, the quantum transition occurs at the “point” of intersection of the electronic terms of reagents and products,^18^ which is achieved as a result of the classical reorganization of the nuclear subsystem, and each individual donor-acceptor pair immersed in the medium acts, as in the case of transitions in individual molecules, as a simple quantum-classical system. An attempt to formalize the qualitative picture of Marcus in the framework of quantum mechanics, which we essentially undertook at the initial stage of building quantum-classical (dozy-chaos) mechanics, leads to a singularity in the rates of transitions (transfers). Damping this singularity, that is, introducing chaos into the transient state of each individual donor-acceptor pair, we obtain the quantum-classical (dozy-chaos) mechanics of elementary electron transfers in condensed matter, which is formulated in Section 2.4. The result of the rate constant in Marcus' theory is obtained from the result of the rate constant in dozy-chaos mechanics when we turn to strong chaos [11] (γ≥E; see also the limiting transition in Section 2.4.9). It follows that in the problem of elementary electron transfers, the Franck-Condon principle acts as an effective simulator of strong dozy chaos, just as in the problem of “quantum” transitions in molecules, and the Marcus model acts as an effective simulator of quantum-classical mechanics of electron transfers in strong dozy chaos.

### 2.6 Explanation for “pedestrians” why quantum mechanics ceases to work in molecular and chemical physics

Quantum mechanics was invented in the last century to explain the quantum jumps of an electron in an atom. Let's assume that the atom can be schematically modeled by a one-dimensional potential box (Fig. 4*a*). Then, what is the model of a molecule in this scheme [49]? In a molecule in the process of a quantum transition from one state to another state, the nuclear subsystem is reorganized, that is, the equilibrium positions of the nuclear vibrations are adjusted to the new distribution of the electron charge in the final state. What does this adjustment or reorganization correspond to in the scheme of the one-dimensional potential box [49]? Obviously, this reorganization corresponds to the motion of at least one of the two walls of the potential box during the quantum transition. There are only two options of the motion of this wall chosen by us. The first option: the wall moves freely, that is, without friction. The second option: the wall moves with friction [14, 15, 18, 28, 49].

Initially, consider the first option. In the course of the electron transition, for example, from the ground state, where the probability of finding the electron is greatest in the middle of the box, to the first excited state, where this probability becomes larger both near the stationary and movable walls, the electron pressure on the walls arises. This pressure sets the free wall in motion. Due to the very rapid motion of the free wall, the width of the potential box increases very rapidly and unlimitedly. As a result, the energy level of the excited state falls very rapidly straight down to coincidence with the energy level of the ground state; this obviously leads to a singularity (to infinity) in the quantum transition rate [14, 15, 18, 28, 49]. From this schematic analysis it follows that, within the framework of quantum mechanics, the full-fledged joint motion of an electron and nuclei in a molecule can not be regular, but it can only be singular. (In the adiabatic approximation [53] (see Section 2.3) in molecular physics and quantum chemistry, the motion of electrons is not dynamically full-fledged: they only form an electric potential in which the nuclei make their oscillations.)

Let us now turn to the second option, in which the wall of the potential box moves with friction. In this case, in the process of a quantum transition, because of the presence of friction, the wall will not have time to go to infinity, and therefore the rate of the electron transition becomes a normal finite value. In a real molecule, this friction corresponds to chaos, which provokes a very light electron in the oscillatory movements of massive nuclei “with the aim” to control their movements during molecular quantum transitions. As previously mentioned (Sections 1, 2.1, and 2.4.3), this chaos is called dozy chaos [14, 28], since it arises merely in a transient state and is absent in the initial and final states. Because of chaos (dozy chaos) in the transient dynamic state, a continuous spectrum of energy in this state appears (see also Sections 1 and 2.1), which is a sign of classical mechanics [16, 18]. In other words, the whole theory of molecular quantum transitions ceased to be quantum mechanics.

Consider the issue in more detail. So, the singularity in the rate constants of electron–nuclear(–oscillatory) transitions, exemplified here by elementary electron transfers in condensed matter, can be elucidated by a potential box with a moveable wall (Fig. 4*a*) [14, 15, 18, 28, 49]. The wall is attached to the abscissa axis by an easily moveable hinge and can shift along the axis with some friction or without friction. Suchlike a wall imitates the nuclear reorganization of the environment in the elementary electron transfers, where dozy chaos acts as friction. In the theory [10, 11, 12, 13, 14, 15, 28, 79], this results in the displacement of the dozy-chaos dependent optical absorption band to the red spectral region and its narrowing (Fig. 4*b*). The width and the intensity of the optical band (see Fig. 4) are controlled by the proportion $\theta_{0}=E/\gamma$ (see Eq. (146)), where E is the reorganization energy and γ is the dozy-chaos energy. The smaller the magnitude of γ is, the sharper “spike” in the dynamic function $t=t\left(\bar{\Omega },\theta_{0}\right)$ (see Fig. 3, γ<<E or $\theta_{0}>>1$), the higher the level of organization of the elementary electron transfer, and the smaller the width and larger the intensity of the optical band (see Fig. 4). The red maximum drift in Fig. 4*b* can be directly understood by considering the potential box with a moveable wall (Fig. 4*a*). This drift can be also realized from the band shape behavior with the change (decrease) in the nuclear reorganization energy in the standard theory, if the reorganization energy is thought of as a complex quantity, and the dozy-chaos energy γ acts as its imaginary part (see details in Ref. [15]).

Consider the basics of quantum mechanics from another point of view. Molecules and atoms are similar to each other. Their similarity lies in the fact that the structural elements of both microsystems are nuclei and electrons. The main difference between these microsystems is the number of nuclei: atoms are electron-nuclear microsystems that have only one single nucleus, while molecules are electron-nuclear microsystems that generally have a large number of nuclei (at least two). It is this well-known and trivial fact that ultimately serves as the divide between quantum and quantum-classical mechanics. Quantum mechanics was created at one time to describe the electronic structure of atoms and quantum transitions of electrons between different states of this structure. In stationary states of atoms, due to the massive and single nucleus, the electronic structure is formed as a result of the movement of electrons in the electric field of this single nucleus. In stationary states of molecules, compared with atoms, due to the presence of several massive nuclei, the dynamic roles of electrons and nuclei change places. Here, not the only heavy nucleus, being “turned off” from the full-fledged dynamics of the entire atomic system, is the “main” source of the electric field that forms the electronic structure of the microsystem. In molecules, such a “main” source of the electric field is the subsystem of light valence electrons, which creates an adiabatic potential that forms the nuclear structure of the microsystem and in which the nuclei make their oscillatory movements. In other words, in the adiabatic approximation, these electrons themselves are “turned off” from the full-fledged dynamics of the entire molecular system. It is this fact of “switching off” in the adiabatic approximation of the valence electron subsystem from full-fledged dynamics that is the condition for the applicability of quantum mechanics to molecular systems. Going beyond the adiabatic approximation leads to the need to consider the full-fledged dynamics of not only nuclei, but also valence electrons. However, due to the incomparability of their masses, in the framework of quantum mechanics this joint dynamics, as shown by the author on the example of elementary electron transfers in condensed matter, becomes singular. In other words, in molecular physics, quantum mechanics works only within the framework of the adiabatic approximation and ceases to work when it goes beyond its framework. The same statement applies to chemical physics, which studies the dynamics of elementary chemical reactions, or in the simplest case, the dynamics of elementary electron transfers in condensed matter, which we consider in this article.

The aforementioned singular dynamics arises, for example, in the process of transitions between the adiabatic ground and first excited states of molecules, as a result of “an attempt by light electrons to reorganize the structure of a very heavy nuclear subsystem only within the framework of quantum mechanics”, so that the structure of the nuclear subsystem would adapt to a new electron charge distribution in an excited adiabatic (or nearly adiabatic) state. The presence of a singularity in the rates of quantum transitions in molecular quantum mechanics is also indicated by the internal inconsistency of the Franсk-Condon principle, which was discussed above in Section 2.5 and which is associated with the inability to give a mathematical description of the qualitative, and essentially quantum-classical, Franck-Condon picture of the dynamics of a molecular transient state. Therefore, in theoretical physics, the discussed singularity must be damped, which was done by the author on the simplest example of elementary electron transfers in condensed matter [10, 11, 12] by introducing chaos (dozy chaos) into the quantum dynamics of the transient state. However, as a result of such damping by introducing chaos, the energy spectrum of moving electrons and nuclei in the transient state becomes continuous [16, 18]. This means that the proposed new dynamic theory is no longer quantum mechanics, but quantum-classical mechanics. Quantum states are the initial and final states in the adiabatic approximation, which are quite sharply different in the electron-nuclear structure, and the transient state is similar to the classical state. The discovery of a new dynamic theory — quantum-classical mechanics, means the discovery in physics of a new fundamental property of an electron. This property appears in an electron as a result of its binding of atomic nuclei into molecular systems and consists in its ability to provoke the necessary chaos in the transient dynamic state of these systems. This new property of an electron makes it possible to eliminate the discussed singularity in the quantum mechanics of molecules and, as a result, to ensure the very possibility of “quantum” transitions in molecular systems. Thus, an electron, being exclusively a quantum microparticle in atoms, where it makes quantum jumps between discrete energy levels, in molecules and condensed matter it becomes a quantum-classical microparticle with a continuous spectrum of energy in the transient state [15, 16, 18, 35, 36, 48, 49].^19^

### 2.7 Dozy chaos and quanta: analogy being in their discovery in physics and perhaps in life

It follows from the previous sections that dozy chaos is introduced into theoretical physics as a new physical substance for eliminating the singularity in the rate constants of quantum transitions which occurs in molecular quantum mechanics when going beyond the adiabatic approximation. In theoretical physics, a similar procedure for introducing a new physical substance, namely, quanta, was carried out at the very beginning of the last century to eliminate the singularity in the distribution function of blackbody radiation at high frequencies. As is known from the history of physics, this singularity is often referred to as an ultraviolet catastrophe. Let me remind you the reason for ultraviolet catastrophe. Classical physics assumes that each mode of blackbody radiation, regardless of its frequency, has on average the same amount of energy, namely energy (by $k_{\text{B}}T/2$ for the electric and magnetic wave energy). In fact, the average energy per mode $\bar{\varepsilon }$ depends on its frequency Ω and decreases exponentially with increasing Ω:

(164) $$\bar{\varepsilon }=\frac{\hbar \Omega }{\exp\left(\hbar \Omega /k_{\text{B}}T\right)-1}\text{,}\;\left(\hbar \to 0,\;\bar{\varepsilon }=k_{\text{B}}T\right)\text{.}$$

**Fig. 5:**
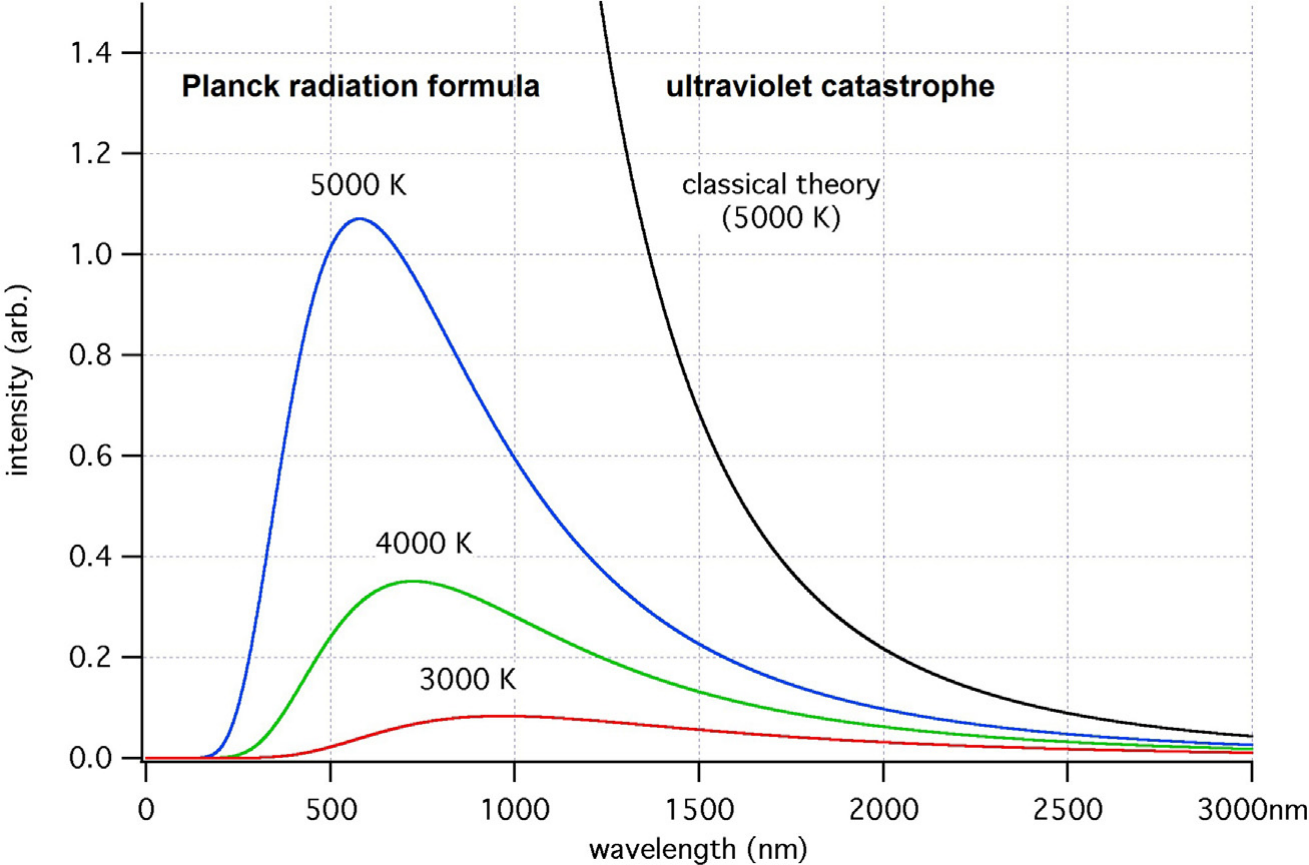
Distribution function of black light ϕ(λ,T) (Eq. (165)).

This fact leads to the elimination of the singularity in the distribution function of black light

(165) $$f\left(\Omega ,T\right)=\frac{\hbar \Omega^{3}}{4\pi^{2}c^{2}}\frac{1}{\text{exp}\left(\hbar \Omega /k_{\text{B}}T\right)-1}\text{,}\;\phi \left(\lambda ,T\right)=\frac{2\pi c}{\lambda^{2}}f\left(\frac{2\pi c}{\lambda },T\right)$$

(see Fig. 5) and, as first shown by Planck [95], is a consequence of the fact that the absorption and emission of energy of electromagnetic waves is not continuous, but occurs in the form of quanta ε=ℏΩ.

The hypothesis of quanta was conflicting to classical mechanics, and in due time, it was entirely beyond the conventional physical concept, but implications of the discovery of quanta proved to be truly breathtaking. As a matter of fact we can say that quantum mechanics, originating in the hypothesis of quanta, underlies the modern science and technological progress. The quantum-classical mechanics under discussion, based on the hypothesis of dozy chaos, provides an understanding of a series of experimental results in chemistry (see Section 1), which for a long time could not be explained within the framework of the standard quantum mechanics of electron-nuclear motion. From general physical considerations it follows that dozy chaos is the cause not only of molecular quantum transitions, but also of the whole variety of chemical reactions, and as a consequence, it is the physical origin of the evolution of molecular matter, up to the emergence of living matter and the person himself [32b, 33a, 33b]. Therefore, there is every reason to believe that the implications of the discovery of dozy chaos and quantum-classical mechanics in our life will be no less impressive in comparison with the implications of the discovery of quanta and quantum mechanics. From general considerations it follows, for example, that dozy chaos constitutes the physical basis for the effective functioning of the brain, which is the main “receptacle” of dozy chaos in the living organism [32b, 33a, 33b]. Since the onset of cancer is usually associated with damage to the genes in cells, for example, by radiation, then the radiation of dozy chaos of the living brain in its pathological functioning, for example under stress, is one of the main physical causes of the natural onset and development of cancer [32b, 33a, 33b].

### 2.8 Outlook for the account of local vibrations in quantum-classical mechanics

The physical picture of molecular quantum transitions (MQT), which was previously discussed, does not at all exclude the presence of quantum jumps in general in the entire dynamics of MQT, in spite of the fact that these quantum jumps are absent in our theory of elementary electron-charge transfers [36, 96]. In our theory, this absence is due to taking into account only the simplest dynamics of MQT, namely, the dynamics that is associated with the interaction of the electronic transition with non-local phonons of the environment; a more complex dynamics of the interaction of the electronic transition with local vibrations is neglected there.^20^ Taking the latter into account will lead to the account in theory, along with the chaotic dynamics of the transient state of electron-vibrational transitions, as well as vibrational quantum jumps. When, together with the reorganization of the nuclear subsystem, the change in the electronic state during the electron transition to the final state is already completed, but in the molecular system there is still some small energy defect, determined by the energy conservation law, then there will be a rapid quantum jump in the vibrational energy of the nuclei, that being already in the final adiabatic electronic state (more precisely, in a state close to adiabatic, if we are talking about the optical excitation of the molecule), in order to eliminate this energy defect. In other words, the quantum jump or transition in the local vibrational mode in the entire process of a molecular “quantum” transition occurs at its final stage, namely, at the moment of transition of a molecule or molecular system from chaotic (dozy-chaos) dynamics to adiabatic (or nearly adiabatic) dynamics. In the experiment, this effect is manifested in the presence of striped spectra and discrete narrow lines in the electron-vibrational spectra that are well-known from the literature (see Refs. [62, 63, 64]).

Within the framework of quantum-classical mechanics, the well-known small line widths corresponding to local oscillations can easily be justified by an estimate that uses our result for the shape of the optical absorption band corresponding to the case of non-local oscillations (see Eqs. (141), (142), (143), (144), (145), (146), (147), (148), (149), (150), (151), (152), (153), (154), (155), (156), (157), and (158) in Section 2.4.8). We assume that the shape of the optical line for local oscillations is close to the Gaussian form known from the standard theory [72], and which in our theory corresponds to the case when the dozy-chaos energy γ is equal to or of the order of the reorganization energy E: γ≅E. In the standard theory [72], the half-width of the optical line (see Eq. (163) in Section 2.4.9) is determined, in particular, by the reorganization energy $\lambda_{\text{r}}\equiv 2E$ and is calculated from the following equation

(166) $$w_{1/2}=2\sqrt{2\;\ln\;2}\sqrt{2\lambda_{\text{r}}k_{\text{B}}T}\text{.}$$

It follows from Eq. (37), which determines the energy of reorganization $E=\frac{1}{2}\sum_{\iota }\hbar \omega_{\iota }{\tilde{p}}_{\iota }^{2}$, that, other things being equal, its value for one non-local or local mode of oscillations of nuclei is much less than its value for a large number of the modes of oscillations of nuclei interacting effectively with the electronic transition. From this and from Eq. (166), small line widths follow, which must be obtained within the framework of quantum-classical mechanics in the future in the case when only local oscillations of the nuclei are taken into account.

Thus, in the general case (that is, taking local vibrations into account) in our picture, just as in the Franck-Condon picture, MQT occur in two stages. However, while in the Franck-Condon picture the rapid quantum jump (in the electron energy) precedes the slow process of nuclear reorganization, in our picture, on the contrary, the quantum jump (in the vibrational energy of the nuclei) occurs when the reorganization of the nuclei, as a result of the electronic transition, has already been completed. The construction of an adequate mathematical technique for the accurate description of such a physical picture is a separate and rather complex problem, the solution of which will require the collective efforts of the scientific community in the future. Until this solution is obtained, one can continue to use successfully the standard and essentially erroneous Franck-Condon explanation given in the existing literature (see Refs. [62, 63, 64]).^21^

The elementary transfer of electron charge in condensed media, when in theory only non-local phonons are taken into account, traditionally refers to chemical physics. In other words, in this article we have demonstrated, strictly speaking, only the fact that quantum mechanics stops to operate in chemical physics and the need to introduce chaos (dozy chaos) in the transient state of elementary electron transfers. In molecular physics, where local phonons must be taken into consideration in theory, this demonstration, as previously mentioned, has yet to be realized. However, more recently, the emergence of chaos in the problem of excitation of a hydrogen molecule under the influence of a periodic external force was theoretically proved [97].

### 2.9 Quantum-classical mechanics as non-mesoscopic physics

Mesoscopic systems are those that are in between the quantum and classical mechanical states. Therefore, the question arises: “How do quantum-classical mechanics reconcile with mesoscopic physics?”

Mesoscopic physics is a phenomenological approach, that is, such an approach when we build *ad hoc* models to account for quantum effects in macroscopic and classical systems. For example, when we consider dimensional quantization (quantum confinement) in nanoparticles, or when we introduce quantization of classical chaos, we obtain quantum chaos. Various examples of mesoscopic physics problems can be found, for example, in Ref. [98], and, in particular, on quantum chaos and its connection with mesoscopic physics, for example, in Ref. [99].

The author's approach is a movement in the opposite direction: this is when we are trying to solve honestly *a priori* quantum problem for a fairly complex physical system.^22^ In contrast to the phenomenological approach in mesoscopic physics, our approach can be called a microscopic approach. An example of such a microscopic approach is the standard theory of many-phonon processes [72], which describes quantum transitions and spectra of luminescence and optical absorption in F-centers [73, 86, 93]. In this theory, the initial and final states of the system are considered in the adiabatic approximation. Because of using the adiabatic approximation there, it is possible to obtain a result for the rate constants of quantum transitions in the framework of quantum mechanics, which contains a dependence on temperature as a consequence of averaging over the equilibrium (Planck) distribution of the initial states in the “molecular” ensemble, in particular, in the ensemble of F-centers. The reason for the success of this approach in explaining optical bands in F-centers is determined by the fact that the standard theory of many-phonon transitions [72] is essentially a special case of quantum-classical mechanics, the case for strong dozy chaos (see Sections 2.4.9 and 2.5). An attempt to go beyond the adiabatic approximation in quantum mechanics, in the theory of many-phonon transitions, leads to a singularity in the rates of quantum transitions. Expressed in a different way, going beyond the adiabatic approximation in quantum mechanics automatically leads to the theory of many-phonon transitions beyond quantum mechanics. Solving this situation is allowed by the natural damping of the singularity, which is described in detail in Section 2.4. Although this natural and forced damping of the singularity does not return the theory of many-phonon transitions to the realm of quantum mechanics, it does provide a physical (non-singular) result for the optical spectra (Section 2.4.8), and a much more general one than given by the standard theory of many-phonon transitions (Figs. 1, 2, and 4*b*). The reason for going beyond quantum mechanics is, ultimately, to take into account the dynamics of the transient state, which, as it turns out, is chaotic. This chaos in the motion of optical electrons and associated atomic nuclei in the transient state leads to a continuous energy spectrum of the entire “electron + nuclear environment” system for a short time (order ℏ/E) of the transition between its initial and final states. That is to say, for a short transition time, the “electron + nuclear environment” system becomes similar to the classical system. In this case, the initial and final stationary states continue to be quantum states, that is, such states that can be described with sufficient success in the adiabatic approximation. Hence the terminology “quantum-classical mechanics.”

The question can be raised whether the ensemble and with it the temperature, which, as a rule, determine the experimental reality, brings an element of classicality into the quantum system, regardless of whether the chaotic dynamics of the transient state in individual molecules is taken into account. The answer is yes, because in the standard theory of many-phonon transitions, we already obtain the result for the shape of the optical band as a continuous function (as a result of the dispersion of phonon frequencies [72]), rather than discrete lines, as in atomic physics. In this experiment, we have the same shape of the optical band as a continuous function. However, by applying the adiabatic approximation, this result for the shape of the optical band as a continuous function in the standard theory of many-phonon transitions is obtained completely within the framework of quantum mechanics. This fact allows us to consider many-phonon transitions in the standard theory as “purely” quantum transitions, and not as quantum-classical transitions, as we do in the new theory, where classicality already arises in the transient state of each single molecule (each donor-acceptor couple in the case of electron transfers) in the ensemble, and to consider the most standard theory of many-phonon transitions not as a theory belonging to mesoscopic physics, but as a quantum-mechanical theory. Concomitantly, it is essential that taking into account only the ensemble of molecules and the associated temperature to simulate an experimental situation, without introducing chaos into the transient state of each single molecule, does not solve the problem associated with the appearance of a singularity in the transition rates in the case of going beyond the adiabatic approximation. In short, classicality, which is introduced into quantum mechanics by an ensemble of molecules and temperature, is not capable of damping the essential singularity in the transition rates.

The physical nature of the singularity is discussed in this article in several aspects: 1) the incomparability of the masses of electrons and nuclei in the presence of full-fledged dynamics of both nuclei and electrons (Sections 2.1 and 2.4.3); 2) quantum transitions in a potential box with a moveable wall (Section 2.6); and 3) internal inconsistency of the Franck-Condon principle (Section 2.5). For example, consider the aspect due to the internal inconsistency of the Franсk-Condon principle. This internal inconsistency is associated with the impossibility in the framework of quantum mechanics to formalize the qualitative picture of the transition proposed by the Franсk-Condon principle, which assumes that in the adiabatic approximation, the same oscillator behaves like a classical and quantum system in the same transition act. Namely, according to the Franck-Condon principle, in the oscillator there is a classical movement of the nuclei to the turning point at which the quantum transition to a new electronic state occurs. Therefore, the Franck-Condon principle with its quantum-classical paradigm is a precursor to the creation of quantum-classical mechanics with its dozy chaos in the transient state of an individual molecule. In the new theory, which gives new physical results in the cases of weak and medium dozy chaos, the Franck-Condon principle acts as an effective simulator of strong dozy chaos. Thus, the new theory of quantum-classical mechanics is not another section of mesoscopic physics with its phenomenological approach, but a natural generalization of the quantum-mechanical theory of many-phonon transitions [72], where the dozy-chaos energy γ≡0,^23^ in the case of γ≠0, that is, taking into account the chaotic dynamics of the transient state in elementary electron transfers in condensed matter, and also in molecules [65] (see Section 2.8) and supramolecular systems in the future. This is a generalization similar to the broad meaning in which Schrödinger's equation as a postulate in quantum mechanics is a generalization of the Hamilton-Jacobi equation for action in classical mechanics, and in general, quantum mechanics is a generalization of classical mechanics, where Planck's constant ℏ≡0, in the case of ℏ≠0 (see Section 2.7). Comparisons between quantum-classical mechanics and other theoretical approaches, where mixing quantum and classical mechanics is undertaken (see *e.g.* [100, 101, 102]), will be given elsewhere.^24^

### 2.10 Dozy chaos as compared to quantum chaos

In modern mesoscopic physics (see *e.g.* Refs. [98, 99]), there is an idea of the so-called quantum chaos, which has been actively studied, mainly theoretically, for several decades now (see Refs. [103, 104, 105, 106, 107, 108, 109, 110, 111, 112, 113, 114, 115, 116, 117, 118, 119]). Dozy chaos differs fundamentally in physical nature from quantum chaos. The term “quantum chaos” is generally understood to comprise all problems concerning the quantum mechanical behavior of classically chaotic systems [103, 104, 105, 106, 107, 108, 109, 110, 111, 112, 113, 114, 115, 116, 117, 118, 119]. In other words, systems, whose underlying classical dynamics is chaotic due to nonlinear interactions [120, 121, 122, 123], exhibit signatures of the chaos in their quantum mechanics. The quantization of a classical dynamical system with chaos is a restriction on chaos, and hence this quantization leads only to a less pronounced (inferior) chaos. Therefore, Berry [104, 105] urged at one time to speak not of quantum chaos, but of quantum chaology, and Chirikov [107] used the term “quantum pseudochaos”. Nevertheless, the term “quantum chaos” has remained generally accepted (see *e.g.* Refs. [103, 108]). One of remarkable results of the theory of quantum chaos are the Heller's scars [106].

As for dozy chaos, in the case of classical mechanics it simply ceases to exist, since in this case molecular quantum transitions, the dynamics of which dozy chaos determines only, cease to exist. Hence, dozy chaos, which provides a classical character of any transient state in molecular quantum transitions, is a purely quantum phenomenon in physical nature, in the sense that it does not exist by definition, in contrast to quantum chaos originating from classical chaos, in the field of classical phenomena. Such a dualistic nature of dozy chaos — its existence only in the field of quantum phenomena and the functional role being in providing, due to chaos, a classical character of any transient state in molecular quantum transitions — is a generalization (in the case of energy γ≠0) of the wave-particle dualism in quantum mechanics (γ=0), which is associated with the statistical nature of physical meaning of the wave function.

Dozy chaos, as compared to quantum chaos, is not associated with nonlinear interactions in a molecular system: dozy chaos appears in a linear problem (see the linear term $\sum_{\iota }U_{\iota }\left(q\right)p_{\iota }$ responsible for the electron-phonon interaction in the Hamiltonian in Eq. (19)). In addition, in contrast to the “inferiority” of quantum chaos, because of the classical nature of the transient state of molecular “quantum” transitions, dozy chaos, like classical chaos, is a full-fledged chaos.

Thus, while the analogue of quantum chaos is classical chaos, there is no analogue for dozy chaos in the field of classical phenomena. As previously mentioned, dozy chaos is the original and universal physical substance, the universal to the same extent to which the electrons and nuclei themselves, the dynamic interactions of which it provides in any “quantum” transition from one of their bound state to another, are universal.

As is clear from the foregoing, the concept of quantum chaos is constructed, by definition, so as to preserve the standard paradigm of quantum mechanics and not to seek a review of its basis in connection with the inclusion of chaos in quantum dynamics. Vice versa, the concept of dozy chaos is constructed, by definition, in such a way that requires a revision of the basis of quantum mechanics, to incorporate the chaotic and classical in nature dynamics into the transient state of molecular quantum transitions.

Summarizing, we can say that the emergence of quantum chaos, for example, in elementary chemical processes, is theoretically considered within the framework of quantum mechanics [112, 113, 114, 115, 116, 117, 118, 119], and quantum chaos becomes a classical chaos when the transition from quantum mechanics to classical mechanics occurs. (Note that in this case of elementary chemical processes with quantum chaos, no clear explanations for or comparisons with the experiment were discussed.) In other words, quantum chaos in quantum mechanics is some analog of classical chaos in classical mechanics. In contrast, there is no analogue of dozy chaos, which is associated with the specific property of an electron to provoke chaos in the vibrations of nuclei, in classical mechanics. In other words, dozy chaos is a “purely quantum” phenomenon related to the specific property of a dynamically active electron in a molecule. We also note that the emergence of quantum and/or classical chaos is associated, as is well-known, with some nonlinear interactions in physical systems, whereas the emergence of dozy chaos occurs in a system with a linear interaction. More detailed comparisons between dozy chaos and quantum chaos will be given elsewhere.

### 2.11 Mathematical techniques and perspectives in quantum-classical mechanics

The formal mathematical techniques are discussed in this section. It is well-known that the standard theory of many-phonon transitions [72] extensively uses the following techniques [11, 12, 14, 28, 79]: the generating polynomial (generating function) technique of Krivoglaz and Pekar [76, 77], which is similar to the Darwin-Fowler method [72, 78] from statistical physics (see Section 2.4.3, footnote 9), the operation calculus of Feynman and Lax [94, 124], the density matrix technique of Kubo and Toyozawa [125, 126], and the quantum field theory technique (see Ref. [72]). Among all methods, the simplest and physically transparent method is the technique of generating functions of Krivoglaz and Pekar. The simplicity and transparency of this technique is due to the fact that it is formulated in terms of the wave function. However, this technique takes into account only the main effect of the electron-phonon interaction — shifts of normal phonon coordinates. The change in the phonon frequencies during electronic transitions and other less significant effects can be taken into account on the basis of the remaining aforementioned techniques. Since the processes of elementary electron-charge transfers in condensed matter and other electron-phonon transitions, in which the dynamics of the transient state must be taken into account, are much more complicated than the standard case of electron-phonon transitions, it is obvious that a generalization of the standard theory of many-phonon transitions should be started, using the simplest technique of Krivoglaz and Pekar. In my technique [11, 12, 52, 75, 79] that generalizing the technique of Krivoglaz and Pekar, as previously shown (Section 2.4.6), all of the intermediate electron-phonon states arising during elementary electron-charge transfers are precisely taken into account. The use of a more sophisticated formal technique, for example, the technique of Feynman path integrals [127], would greatly complicate the whole problem rather than simplify it. I believe that the next main stage in the development of the quantum-classical mechanics of elementary electron transfers [11, 12, 15, 79] will be based on a generalization of the Kubo-Toyozawa density-matrix technique or the quantum field theory technique.

### 2.12 Quantum-classical mechanics and quantum information

This section discusses once again the role of dozy chaos in the elimination of the aforementioned singularity in molecular quantum transitions [15, 34]. In this section, we want to emphasize the fact, which is associated with the elimination of this singularity, that the chaotic nature of the interaction of very light electrons and heavy nuclei in the transient state of molecular “quantum” transitions is the reason for their self-organization. As a result of electron provocation of chaos in the vibrational motion of nuclei, part of the vibrational motion is transformed into the translational movement of their equilibrium positions to new positions that correspond to a new distribution of electron charge. In the process of completing the formation of this new distribution of the electron charge in the final state, the resulting translational motion of the nuclei is transformed back into their vibrational motion, but near the new equilibrium positions.

Thus, as the driver of the reorganization process of the nuclear subsystem in a molecule in addition to the electronic motion, which by creating chaos starts the process, the very vibrational motion of the nuclei acts, which is transformed into a chaotic motion, performing in effect the very process of reorganization of the nuclei. (Vibrations of the nuclei in a molecule are always present, even at absolute zero and zero-point oscillations of the nuclei.) Moreover, the motion of the electrons and nuclei in a molecule in the process of a quantum transition (for example, excitation of an electron-vibrational transition) is undergoing a dramatic change in its nature. Namely, the quantum motion in the adiabatic ground state dies as a result of the launch of the chaotic process of a “quantum” transition and again revives as a result of the completion of this process in the excited adiabatic (or nearly adiabatic) state.^25^ The motion of the electrons and nuclei in the transient state of a molecule has a paradoxical classical nature at first glance.

Above, we described a new mechanism of electron-vibrational transitions as if the electron has free will. In fact, we are discussing the dynamic self-organization of complex molecular systems, which is presented here on the simplest example of the dynamic self-organization of electron-vibrational transitions in molecules. Similar issues are widely discussed at present in such fields of physics as quantum information and cybernetic physics (see Refs. [128, 129, 130, 131, 132, 133, 134, 135, 136, 137, 138, 139, 140] and references therein). In a chaotic molecular transient state, there is essentially an intense exchange of information between a very light electron and heavy nuclei about their current energies and states of motion. The consequence of this exchange of information is the regular, but not singular, dynamics of the transient state [14, 28].

## 3 Conclusions

This article focuses on the regular exposition of dozy-chaos mechanics or quantum-classical mechanics of elementary electron transfers in condensed matter, which are the simplest cases of molecular quantum transitions. For the theoretical description of molecular quantum transitions, quantum-classical mechanics arises in molecular and chemical physics instead of quantum mechanics as a consequence of a critical examination of the Franck-Condon principle, widely known and popular in optical molecular spectroscopy, which was introduced in optical spectroscopy as an external addition to quantum mechanics almost 100 years ago. Quantum-classical mechanics allows, for example, carrying out a theoretical examination of the shapes of optical bands in polymethine dyes and their aggregates, which can only be explained if the Franck-Condon principle is abandoned in principle. According to the arguments given in this article, the Franck-Condon principle remains to date an unsubstantiated hypothesis, despite its successful application to explain a substantial number of experimental facts. The dynamics of the molecular quantum transition, which is given by the Franck-Condon principle, does not stand up to criticism on the basis of general physical considerations (see Sections 2.1 and 2.5). The main critical circumstance is the fact that up to the present, in the framework of quantum mechanics, there is no distinct quantitative solution to the problem of the dynamics of the molecular transient state, at least in some simplest cases, which would confirm the Franck-Condon principle and/or the Franck-Condon dynamics of the transient state. Moreover, the study of the dynamics of the molecular transient state has a physical meaning only when going beyond the Born-Oppenheimer adiabatic approximation, since in this approximation, the full-fledged dynamic role of the electronic subsystem is absent, and the role of the electronic subsystem is reduced solely to the formation of the adiabatic potential in which the nuclei move. However, as shown by the author in the example of a new theory of elementary electron transfers in condensed matter, going beyond the adiabatic approximation leads to a singularity in the rate constants of “quantum” transitions (see Section 2.4.3), which agrees with the general qualitative physical considerations in Section 2.1. At a qualitative level, a vivid physical demonstration of this singularity is given in Section 2.6 in the example of a potential box with a moveable wall. Therefore, to correctly consider the dynamics of the molecular transient state, this singularity must be damped. In other words, we are forced to introduce into molecular quantum mechanics some additional assumption or postulate that this damping would provide, similar to how quantum mechanics was in due time forced to be supplemented from the outside by the Franck-Condon principle.

In the example of the problem of elementary electron transfers in condensed matter, the specified singularity is easiest to dampen if the infinitesimal imaginary additive in the energy denominator of the spectral representation of the total Green's function of the system is replaced by its finite value. In other words, for the first time the problem of the dynamics of a molecular transient state was solved quantitatively by the example of the problem of elementary electron transfers in condensed matter, that is, the solution to the problem was found as discussed in Section 2.1. A successful quantitative explanation, based on this new theory, of the basic set of experimental data on the shape of the optical bands in polymethine dyes and their aggregates showed that the absolute value of the new imaginary additive in the energy denominator of the total Green's function of the system significantly exceeds the magnitude of the quantum of nuclear vibrations. This additive could be interpreted as the energy width of the electron-vibrational virtual levels of the transient state, which provides a multiple exchange of motion and energy between different vibrational modes and the electron in the transient state. However, because of the aforementioned excessively large value of this additive, the exchange of motion and energy between different vibrational modes and the electron in the transient state proves to be so intense that it leads to chaos both in the motion of the medium nuclei and in the motion of the electron that undergoes an elementary transfer. Moreover, from the physical perspective, this can be interpreted in such a way that it is the electron, as very light and mobile, rather than the surrounding nuclei, that provokes chaos in a transient state. The electron provokes chaos to be able to easily control the movement of very heavy nuclei in a transient state. This involves the discovery of a new and universal property of an electron, namely, the property of creating chaos in the motion of nuclei in a transient state during a molecular quantum transition. Electrons acquire this universal property when they bind atomic nuclei to molecules. This is called dozy chaos, since it is not present either in the initial or final states and arises only during molecular quantum transitions. The presence of strong chaos (dozy chaos) in a transient state easily explains why the Franck-Condon principle is often so effective in applications. In strong dozy chaos, as previously shown using the example of elementary electron transfers, the rate constants of molecular quantum transitions cease to depend on the dynamics of the transient state (see Section 2.6, Fig. 4*b*, where the case of strong dozy chaos corresponds to the blue low-intensity Gaussian-like curve). They depend only on the initial and final adiabatic states of the molecular system, with which the Franck-Condon principle essentially only deals. Apparently, this is why dozy chaos has eluded the attention of researchers for so long.

Since according to the Franck-Condon principle, the same oscillator behaves in the same elementary molecular act both as a quantum system (near the turning points of the nuclei) and as a classical system (near the bottom of the potential well for the nuclei), then the Franck-Condon principle is essentially one of the most important prerequisites for the creation of quantum-classical mechanics (see Sections 2.1 and 2.5), the simplest implementation of which is demonstrated in this article.

When dozy chaos is not strong enough to be simulated by applying the Franck-Condon principle, elements of dynamic self-organization manifest themselves in the chaotic dynamics of the molecular transient state. It is to this case that electronic transitions occur in polymethine dyes and their aggregates in solvents. Taking into account these elements of dynamic self-organization in theory allowed the author to quantitatively explain the optical band shapes in polymethine dyes and their aggregates (Section 1). The dynamic self-organization of the transient state is most clearly manifested in the case of J aggregates, where the elementary transfer of the alternating electron charge along the quasi-linear optical chromophore is facilitated by a quasi-synchronous motion of the environmental nuclei (Section 1). This fact manifests itself in a very narrow and intense optical J band, and in this connection J aggregates are widely used in a variety of applications (see Refs. [14, 28] and references therein). An overview of other cases of successful applications of dozy-chaos mechanics is briefly provided in the introduction to this article (Section 1).

Quantum-classical (dozy-chaos) mechanics of the elementary electron transfers in condensed matter, which was earlier used to quantitatively explain the optical band shapes in polymethine dyes and their aggregates (Section 1), allows for only non-local phonons and neglects local phonons. As a consequence, this leads to a relatively simple transient state with a continuous spectrum of electron-vibrational energies, that is, during molecular “quantum” transition — elementary electron-charge transfer, the quantum system — “electron + environment” — becomes a classical system. This provides (together with the dispersion of phonon frequencies [72]) the continuous optical spectra of polymethine dyes and their aggregates, which were discussed in Section 2.1. Taking into account local phonons is a much more complicated problem than considering non-local phonons, and the solution will require considerable efforts from researchers in the future. This account will also lead to a complication of the transient state, in which, along with the chaotic classical dynamics of the electron-nuclear motion, quantum jumps in the vibrational energy of the nuclei at the final stage of the molecular “quantum” transition appear, that is, after the reorganization of the entire nuclear subsystem has already been completed (see Section 2.8). As a result of this account, in the theoretical optical spectra, along with continuous bands corresponding to non-local phonons, striped spectra appear corresponding to both non-local and local phonons. The dominance of any particular local modes in the molecular system should lead to well-defined discrete and narrow vibrational lines in the theoretical and experimental optical spectra (see Section 2.8).

The methods for calculating the shape of the optical bands in polymethine dyes and their aggregates, based on the quantum classical mechanics of electron transfer, can easily be extended to a wide range of other similar objects studied in organic chemistry, for example, styryl dyes and charge-transfer complexes. Generalization to more complicated cases of quantum-classical transitions than electron transfer, due to the large cumbersomeness of the mathematical apparatus arising here, will require the collective efforts of the scientific community.

To confirm the new physical picture of molecular quantum transitions, based on the concept of dozy chaos, it is of great interest to carry out experiments on its direct detection [15, 18, 33, 34, 35, 36, 96]. In this regard, it would be interesting, for example, to register the loss of regularity of the structure of a molecule in its chaotic transient state [96], using modern X-ray free electron lasers [141].

In conclusion, future research should focus on quantum-classical mechanics and its application to luminescence spectra of J aggregates and their comparison with absorption spectra [142].

## Declarations

### Author contribution statement

All authors listed have significantly contributed to the development and the writing of this article.

### Funding statement

This work was supported by the Ministry of Science and Higher Education within the State assignment Federal Scientific Research Center “Crystallography and Photonics” Russian Academy of Sciences.

### Competing interest statement

The authors declare no conflict of interest.

### Additional information

Data associated with this study has been deposited at Dryad Digital Repository under the accession number https://doi.org/10.5061/dryad.t0r3p.

## Footnotes

1 The simplicity is associated herein with approximating the electron's Green's function by it in the case of a free electron and considering merely non-local vibrations of nuclei and disregarding their local vibrations (see above) [11, 18].

2 One exception is the Lamb shift in atomic spectroscopy [58, 59].

3 Formally, there are no quantum transition dynamics in the Franck-Condon principle. This principle simply assumes that, as the initial and final states enter the matrix element of the quantum transition, the wave functions are considered in the adiabatic approximation. However, this assumption can be interpreted physically as previously indicated.

4 For example, why do nuclei with substantial inertia follow the new distribution of the electron charge rather than returning the excited electron to the initial state? See details below.

5 The usual reasoning is: “It is obvious that the nuclei spend most of their time near the turning points. Therefore, the probability of the electron and ‘intended’ vertical transition near these points will be maximal.”

6 Since the energy of the rotational quantum is much less than the energy of the vibrational quantum, for simplicity, hereinafter, we take into account only the vibrational system of degrees of freedom and ignore the rotational one.

7 In this section and throughout this article, we assign the names of the pioneers of the theory of molecules, namely, the names of Born and Oppenheimer, to the adiabatic approximation, although between the adiabatic theory of Born and Oppenheimer [53] and the modern interpretation of the adiabatic approximation [65, 66, 67], which we cite here, there are some differences, which are discussed, for example, in Refs. [66, 67].

8 The adiabatic approximation is often disturbed even in the ground state, for example, due to vibronic interactions [66, 68, 69] and in non-rigid molecular systems [70, 71].

9 Usually, the quantity γ specifies the rule for bypassing singular points in contour integration. However, in our theory of charge transfers [11], some mathematical technique [52, 75] (see Section 2.4.6) manages to perform an exact summation over the intermediate states that are associated with γ. This “releases γ from former duties” and lets it to gain a new status. The author's mathematical technique [52, 75] is a substantial development of the generating polynomial (generating function) technique of Krivoglaz and Pekar [76, 77], and the Krivoglaz-Pekar technique [76, 77] in its turn is analogous to computing the thermodynamic quantities by the Darwin-Fowler technique [72, 78], known from statistical physics.

10 We note that, in analogy with the standard theory of many-phonon transitions [72], our original Hamiltonian (Eq. (19)) depends on the electronic and normal phonon coordinates of the system, and in solving this problem on its basis it is not clear how exactly, because of the incomparability of the masses of the electron and nuclei, a singularity is “formed” in the observed physical quantities, the emergence of which follows from the above analysis of the problem on a qualitative physical level (see Section 2.1). This singularity manifests itself formally in the observed physical quantities, either as γ tends to zero (γ→0) (see Fig. 4 in Section 2.6) or for γ≡0 in the whole of the original theory.

11 For optical transitions, the perturbation in Eq. (64) is accepted in the regular long-wave approximation: W=p, where p is the momentum operator of the electron (see Ref. [61]).

12 The factor ${\bar{m}}_{1}$ appears from considering the equilibrium distribution of the population for $m_{1}$ initial states of phonons.

13 That is, they coincide with the eigenfunctions of the electronic Schrödinger's Eq. (22), which are obtained in the zero order of the adiabatic perturbation theory of Pekar [72, 73] with respect to the operator (25).

14 Heat energy Δ<0 matches the acts of luminescence, research of which is beyond the scope of this article. See also the latest reference in this article.

15 Inter alia, the restriction (136) here means that we neglect the well-known zero-phonon line (on zero-phonon lines, see Refs. [65, 66, 84]).

16 Condition (145) does not allow us to make this limit in our result (141, 142, 143, 144, 145, 146, 147, 148, 149, 150, 151, 152, 153, 154, 155, 156, 157, 158) as the gamma tends to infinity (γ→∞). However, this condition is not fundamental and is related only to routine simplification of calculations, namely it is connected, as previously indicated, with the expansion of the radical into a series of smallness $\left|\gamma \right|/J_{1}$ in the exponent of the Gamow exponent, which is limited to the zero-order approximation. If this simplification is refused, then the result of the limiting transition under discussion is the same as here: K(γ→∞)→0. In this connection, the rejection of the aforementioned expansion would lead to an unjustified complication of the result, which is significant only in an uninteresting, from the perspective of applications, asymptotic parameter range (that is, in the region K(γ→∞)→0).

17 The incomparability of the masses of electrons and nuclei is present in the Franck-Condon picture and “substantiates” it, but here, first of all, we start in our reasoning from the internal inconsistency of the Franck-Condon picture itself, when the incomparability of the masses of electrons and nuclei recedes into the background.

18 For a system with *M* nuclear coordinates, this intersection occurs on an (*M* – 1)-dimensional surface.

19 We are only talking about the outer electrons that are involved in the creation of chemical bonds between atoms. Internal electrons, like in atoms, are tightly bound by their single nucleus.

20 For non-local vibrations of nuclei (crystal lattice phonons), the many-phonon transitions with phonon occupation numbers changing by over 1 do not contribute to the result for the rate constant of quantum transitions and can be neglected. In the case of localized phonons (local vibrations), the many-phonon transitions with all phonon occupation numbers varying from zero “ad infinitum” make the contribution to the result and hence many of them must be taken into account. About the difference in the interaction of the electronic transition with non-local and local phonons, you can see Section 2.4.7 and a review by Perlin [72].

21 Nevertheless, the erroneous Franck-Condon picture for simulation of the transient state of MQT can continue to be used even after the correct solution of the MQT problem will also be obtained in the general case taking local vibrations into account. For example, in our daily life we successfully use the erroneous idea that every day the Sun rises in the East and sets in the West, although in reality it is not about the motion of the Sun in the sky, but the rotation of the Earth around its axis. However, when we go beyond the surface of the Earth into Space, the use of the correct physical picture becomes absolutely necessary. In the simplest case of MQT, this “entry into Space” corresponds to the construction of the theory of the shape of the optical bands in polymethine dyes and their aggregates (see Section 1).

22 An *a posteriori* quantum-classical problem.

23 More precisely, in quantum mechanics energy γ is an infinitely small value.

24 As a rule, these theoretical approaches do not demonstrate clear applications to the experiment.

25 The remark in parentheses does not apply, for example, to elementary electron-charge transfers or elementary chemical reactions, in which “quantum” transitions from one adiabatic ground state to another adiabatic ground state take place.
